# Mouse Liver Compensates Loss of Sgpl1 by Secretion of Sphingolipids into Blood and Bile

**DOI:** 10.3390/ijms221910617

**Published:** 2021-09-30

**Authors:** Anna Katharina Spohner, Katja Jakobi, Sandra Trautmann, Dominique Thomas, Fabian Schumacher, Burkhard Kleuser, Dieter Lütjohann, Khadija El-Hindi, Sabine Grösch, Josef Pfeilschifter, Julie D. Saba, Dagmar Meyer zu Heringdorf

**Affiliations:** 1 Institut für Allgemeine Pharmakologie und Toxikologie, Universitätsklinikum, Goethe-Universität Frankfurt am Main, Theodor-Stern-Kai 7, 60590 Frankfurt am Main, Germany; spohner@em.uni-frankfurt.de (A.K.S.); jakobi@med.uni-frankfurt.de (K.J.); pfeilschifter@em.uni-frankfurt.de (J.P.); 2 Institut für Klinische Pharmakologie, Universitätsklinikum, Goethe-Universität Frankfurt am Main, Theo-dor-Stern-Kai 7, 60590 Frankfurt am Main, Germany; trautmann@med.uni-frankfurt.de (S.T.); thomas@med.uni-frankfurt.de (D.T.); El-Hindi@med.uni-frankfurt.de (K.E.-H.); groesch@em.uni-frankfurt.de (S.G.); 3 Institut für Pharmazie, Pharmakologie und Toxikologie, Freie Universität Berlin, Königin-Luise-Straße 2-4, 14195 Berlin, Germany; fabian.schumacher@fu-berlin.de (F.S.); burkhard.kleuser@fu-berlin.de (B.K.); 4 Institut für Klinische Chemie und Pharmakologie, Universitätsklinikum Bonn, Sigmund-Freud-Straße 25, 53127 Bonn, Germany; Dieter.Luetjohann@ukbonn.de; 5 Department of Pediatrics, Division of Hematology/Oncology, University of California, 505 Parnassus Ave, San Francisco, CA 94143, USA; Julie.Saba@ucsf.edu

**Keywords:** sphingosine-1-phosphate, SGPL1, ceramides, cholesterol, liver, bile

## Abstract

Sphingosine 1 phosphate (S1P) lyase (*Sgpl1*) catalyses the irreversible cleavage of S1P and thereby the last step of sphingolipid degradation. Loss of *Sgpl1* in humans and mice leads to accumulation of sphingolipids and multiple organ injuries. Here, we addressed the role of hepatocyte *Sgpl1* for regulation of sphingolipid homoeostasis by generating mice with hepatocyte-specific deletion of *Sgpl1* (*Sgpl1*^HepKO^ mice). *Sgpl1*^HepKO^ mice had normal body weight, liver weight, liver structure and liver enzymes both at the age of 8 weeks and 8 months. S1P, sphingosine and ceramides, but not glucosylceramides or sphingomyelin, were elevated by ~1.5–2-fold in liver, and this phenotype did not progress with age. Several ceramides were elevated in plasma, while plasma S1P was normal. Interestingly, S1P and glucosylceramides, but not ceramides, were elevated in bile of *Sgpl1*^HepKO^ mice. Furthermore, liver cholesterol was elevated, while LDL cholesterol decreased in 8-month-old mice. In agreement, the LDL receptor was upregulated, suggesting enhanced uptake of LDL cholesterol. Expression of peroxisome proliferator-activated receptor-γ, liver X receptor and fatty acid synthase was unaltered. These data show that mouse hepatocytes largely compensate the loss of *Sgpl1* by secretion of accumulating sphingolipids in a specific manner into blood and bile, so that they can be excreted or degraded elsewhere.

## 1. Introduction

Sphingolipids are in the focus of current research because they are part of fundamental cellular processes (for a recent review, see [1]). De novo synthesis of sphingolipids leads to formation of ceramides, which can be metabolized reversibly to sphingomyelin, glycosphingolipids, or sphingosine. Sphingosine can be converted back to ceramides by ceramide synthases (CerS), or phosphorylated reversibly to sphingosine-1-phosphate (S1P) by sphingosine kinases (SphK) [1]. S1P lyase (SGPL1) catalyzes the irreversible cleavage of S1P which is the only pathway for degradation of the sphingoid base backbone (reviewed in [2]). S1P is an important bioactive lipid that acts both as agonist at five specific G-protein-coupled receptors (GPCR) and intracellularly (reviewed in [3]). 

The S1P-GPCR, S1P_1-5_, couple differentially to G_i_, G_q_, and G_12/13_ proteins and thereby regulate cell proliferation, survival, migration, adhesion, and other responses (reviewed in [4]). They are involved in vertebrate development, immune cell trafficking, regulation of vascular tone and permeability, and homoeostasis of many tissues (reviewed in [3]). S1P-GPCR are important pharmacological targets because of their pathophysiological role in autoimmunity, inflammation, fibrosis, and cancer [5]. Intracellular activities of S1P are not well understood but it has been suggested that intracellular S1P inhibits histone deacetylases, regulates mitochondrial respiration by binding to prohibitin-2, stabilizes telomerase reverse transcriptase, and more (discussed in [3]). Cellular export of S1P occurs via spinster-2, major facilitator superfamily domain-containing protein-2B (Mfsd2b), or several ATP-binding cassette (ABC) transporters [6,7,8]. Plasma S1P is derived mainly from endothelial cells and erythrocytes, and bound to albumin or to apolipoprotein M (ApoM) within HDL (reviewed in [9]). S1P lyase and S1P transporters contribute to formation of S1P gradients between plasma and tissues and within tissues, which guide immune cell trafficking and cell positioning (reviewed in [10]).

The importance of S1P lyase becomes obvious when looking at patients with loss-of-function *Sgpl1* mutations. These patients suffer from S1P lyase insufficiency syndrome (SPLIS), which comprises steroid-resistant nephrotic syndrome, primary adrenal insufficiency and neuropathy [11,12,13]. Other symptoms of SPLIS, with variable preponderance, may be lymphopenia, immunodeficiency, endocrine defects, skin alterations, and dyslipidemia (reviewed in [14]). Human SPLIS is mimicked in many aspects by deletion of *Sgpl1* in mice. Global *Sgpl1* knockout mice suffer from growth retardation, reduced life span, and multiple organ defects, including kidney damage, neurodevelopmental delay, glucocorticoid deficiency, and malformations of vasculature and skeleton [15,16,17]. Mice with constitutive or inducible *Sgpl1* knockout had elevated levels of S1P and sphingosine in blood and tissues [18,19] and also ceramides and sphingomyelin were elevated in serum of constitutive knockout mice [18]. Disruption of the blood-tissue S1P gradient led to lymphopenia [16]. Furthermore, mice with global *Sgpl1* knockout had a pro-inflammatory phenotype with neutrophilia, elevated liver and kidney cytokines, and impaired recruitment of neutrophils into inflamed tissues [17,20]. Inducible *Sgpl1* knockout mice had markedly increased bone mass and strength, combined with less adipose tissue, due to activation and inhibition of the transcription factors, osterix and Pparγ, respectively [21]. Finally, brain-specific knockout of *Sgpl1* showed its importance for normal presynaptic architecture [22] and cognitive skills [23].

Global *Sgpl1*-deficient mice have a distinct liver phenotype with increased levels of S1P, sphingosine, ceramides and sphingomyelin, along with accumulation of phospholipids, triglycerides, diacylglycerol, and cholesterol [18]. Total and free cholesterol, cholesterol esters as well as very low density lipoprotein (VLDL), low density lipoprotein (LDL), and high density lipoprotein (HDL) cholesterol were also highly elevated in the serum of these mice [18]. Mechanistically, the authors suggested an involvement of *Pparγ* which was found to be induced [18]. We have recently shown another link between SGPL1 and cholesterol homoeostasis in embryonic fibroblasts from *Sgpl1*-deficient mice, in which cholesterol was trapped in endo/lysosomes [24]. This led to activation of sterol regulatory element-binding protein-2 (SREBP2), subsequent upregulation of the LDL receptor, and enhanced uptake of cholesterol. These and other features such as upregulation of the amyloid precursor protein (APP) and increase in certain oxysterols were reminiscent of Niemann-Pick disease type C (NPCD) [24]. In NPCD, mutated NPC-1 and NPC-2 proteins fail to deliver cholesterol from endo/lysosomes to the endoplasmic reticulum, interestingly causing a secondary accumulation of sphingolipids (see [25]).

Aim of the present study was to characterize the role of hepatocyte *Sgpl1* for regulation of sphingolipid metabolism and cholesterol homoeostasis. We show here that mice with hepatocyte-specific *Sgpl1* knockout (*Sgpl1*^HepKO^) did not differ much from control mice from the same breed (*Sgpl1*^control^). Interestingly, very long chain ceramides were elevated in plasma, and S1P and glucosylceramides were elevated in bile of *Sgpl1*^HepKO^ mice, indicating that mouse liver effectively eliminated excess sphingolipids via secretion into blood and bile.

## 2. Results

**Figure 1:**
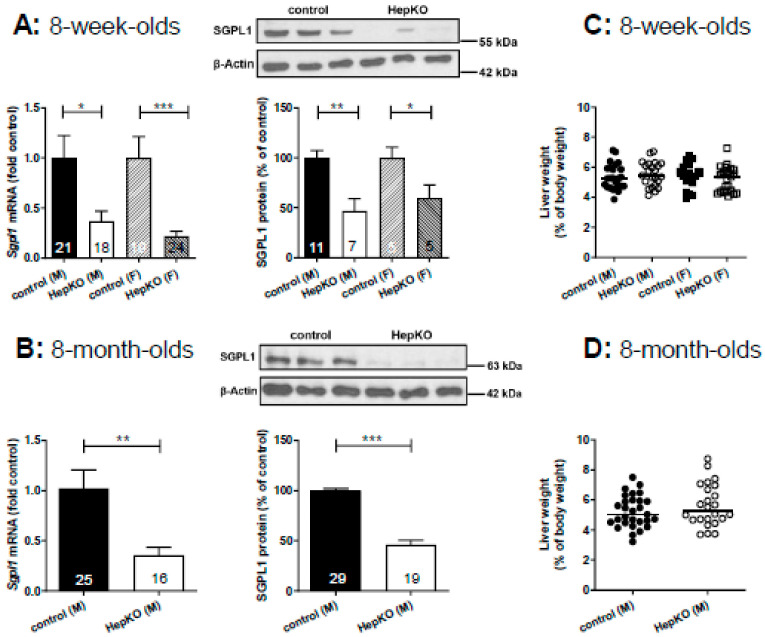

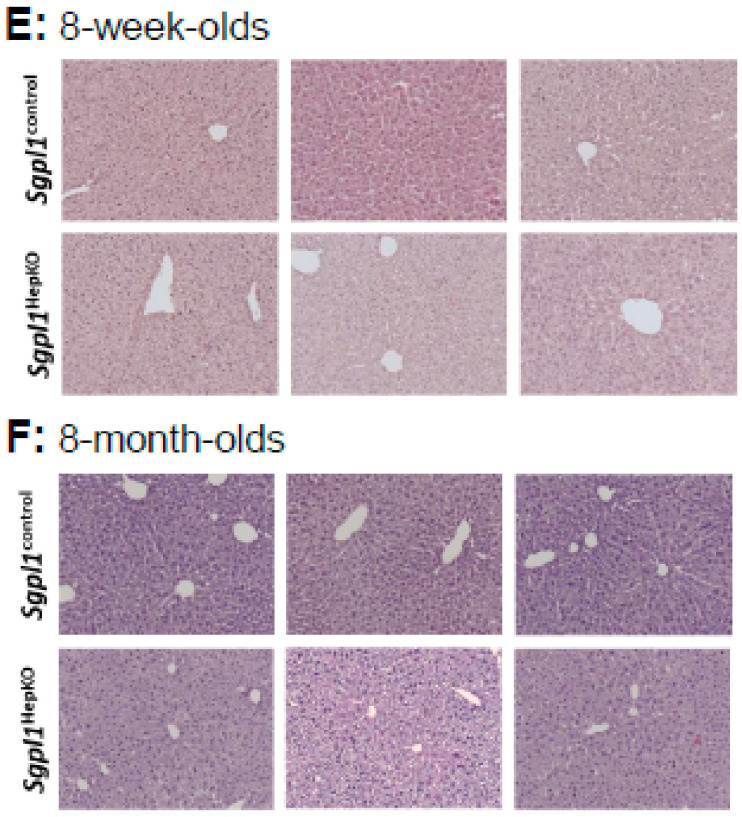
Characteristics of *Sgpl1*^HepKO^ mice. *Sgpl1* mRNA and SGPL1 protein levels were determined by quantitative real-time PCR and Western blotting, respectively, in liver homogenates of *Sgpl1*^control^ and *Sgpl1*^HepKO^ mice at the age of 8 weeks (**A**) or 8 months (**B**). The presented Western blots show representative samples from male mice; please note that two different molecular weight markers have been used in A and B. All values are means ± SEM; annotations within the bars indicate the numbers (*n*) of individual mice. * *p* < 0.05, ** *p* < 0.01, *** *p* < 0.001 in nonparametric Mann-Whitney test (mRNA) or two-tailed Student’s *t*-test (protein). (**C**,**D**) Liver weight expressed as % of body weight of mice at the age of 8 weeks (**C**, *n* = 19–27) or 8 months (**D**, *n* = 29 and 24). Shown are values of individual mice and their median. (**E**,**F**) H&E staining of liver slices prepared from *Sgpl1*^control^ and *Sgpl1*^HepKO^ mice. Shown are representative slices from male mice (3 of each group). M, male mice; F, female mice.

In livers of *Sgpl1*^HepKO^ mice at the age of 8 weeks, expression of *Sgpl1* mRNA was reduced by ~60% (males) and ~75% (females) when compared to *Sgpl1*^control^ mice from the same breed (Figure 1A). SGPL1 protein was reduced by ~50% (males) and ~40% (females) (Figure 1A). Similarly, 8-month-old male *Sgpl1*^HepKO^ mice had a significantly reduced *Sgpl1* mRNA (~60%) and SGPL1 protein (~50%) expression (Figure 1B). Thus, SGPL1 was significantly suppressed in livers of *Sgpl1*^HepKO^ mice and this was stable during aging. The remaining mRNA and protein expression is probably due to expression of SGPL1 by cells other than hepatocytes.

8-week-old *Sgpl1*^HepKO^ mice were normal with respect to body and liver weight (Figure S1A), and liver weight as % of body weight (Figure 1C). Liver enzymes in blood, alanine aminotransferase (ALT) and aspartate aminotransferase (AST) were not significantly altered (Figure S1C). In lipid storage diseases, the phenotype often progresses with age [26]. Therefore, we bred 8-month-old *Sgpl1*^HepKO^ mice. However, despite stable SGPL1 suppression (see above), liver and body weight as well as liver enzymes were still normal compared to age-matched *AlbCre*^−/−^ controls (Figure 1D and Figure S1B,D). Furthermore, *Sgpl1*^HepKO^ mice had a normal liver structure in haematoxylin and eosin (H&E) staining at both 8 weeks and 8 months of age (Figure 1E,F). Since mice with global *Sgpl1* knockout showed lymphopenia and neutrophilia [20], blood cell counts were evaluated. However, numbers of lymphocytes, granulocytes and monocytes were normal in 8-week- and 8-month-old *Sgpl1*^HepKO^ mice (Figure S1E,F).

**Figure 2:**
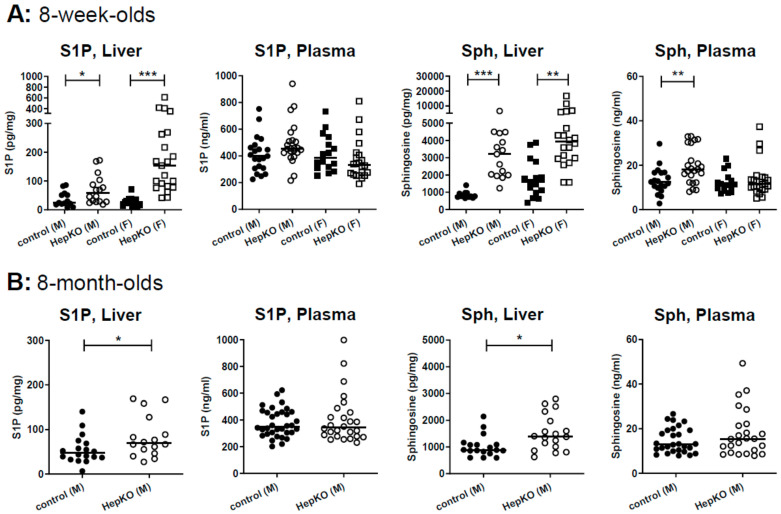
Levels of S1P and sphingosine in liver and plasma of *Sgpl1*^HepKO^ mice. Levels of S1P and sphingosine (Sph) were measured by LC-MS/MS in liver and plasma of *Sgpl1*^control^ and *Sgpl1*^HepKO^ mice at the age of 8 weeks (**A**; *n* = 13–23) or 8 months (**B**; *n* = 16–34). Shown are values from individual mice and their median. * *p* < 0.05, ** *p* < 0.01, *** *p* < 0.001 in two-tailed Student’s *t*-test with Welch’s correction.

LC-MS/MS analysis of S1P and sphingosine revealed that hepatocyte-specific *Sgpl1* deletion led to a significant accumulation of both lipids in the liver of 8-week-old mice (Figure 2A). Of note, plasma S1P was not significantly elevated by hepatocyte-specific *Sgpl1* deletion, while plasma sphingosine was slightly elevated (~1.5-fold) in male but not female *Sgpl1*^HepKO^ mice (Figure 2A). At the age of 8 months, S1P and sphingosine were again elevated in the liver of *Sgpl1*^HepKO^ mice, but in contrast to our expectation, not as much as at the younger age (Figure 2B). Thus, sphingosine levels in liver were increased by 3.8 ± 0.4-fold in 8-week-old but only by 1.5 ± 0.2-fold in 8-month-old *Sgpl1*^HepKO^ mice (means ± SEM; *p* < 0.0001; Figure 2A,B). Importantly, plasma concentrations of S1P and sphingosine were normal in 8-month-old *Sgpl1*^HepKO^ mice (Figure 2B). These results, together with the normal liver weight and structure reported above, suggest that there are mechanisms which compensate for loss of SGPL1 in hepatocytes, and that these compensatory mechanisms are more effective with age.

**Figure 3:**
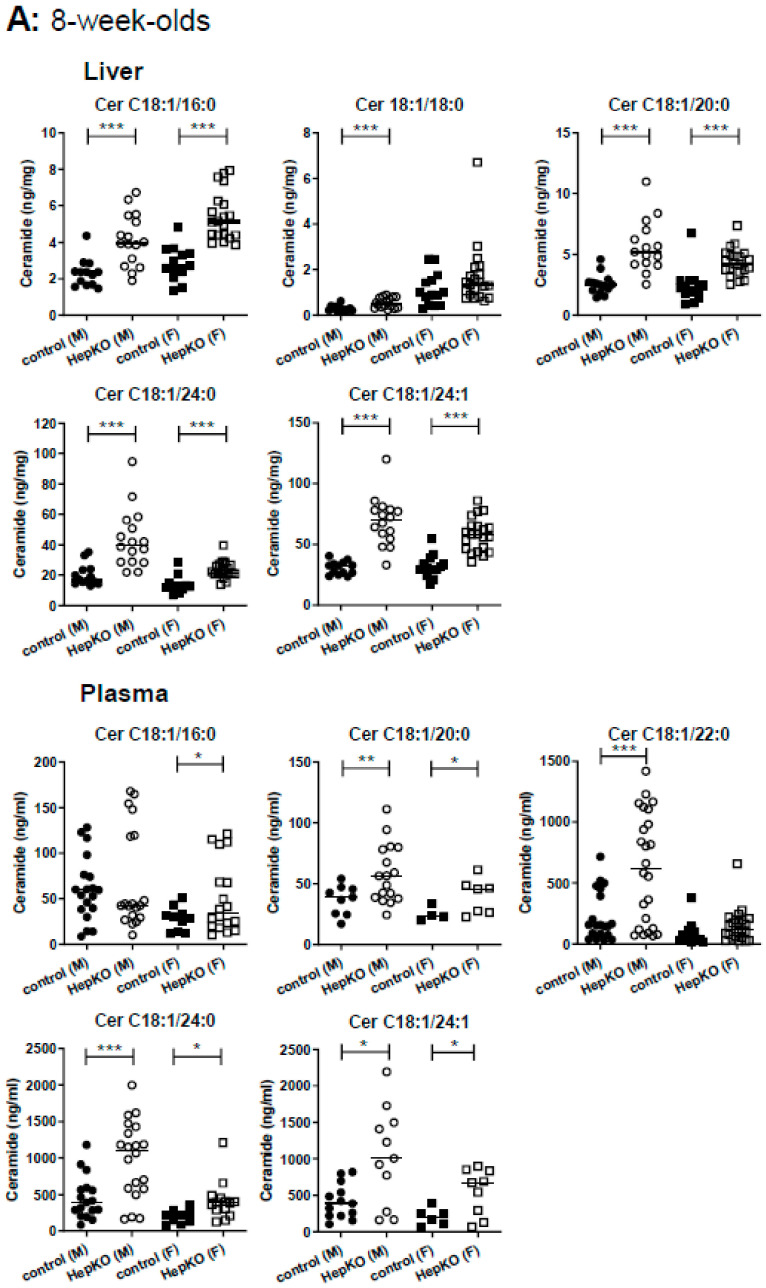

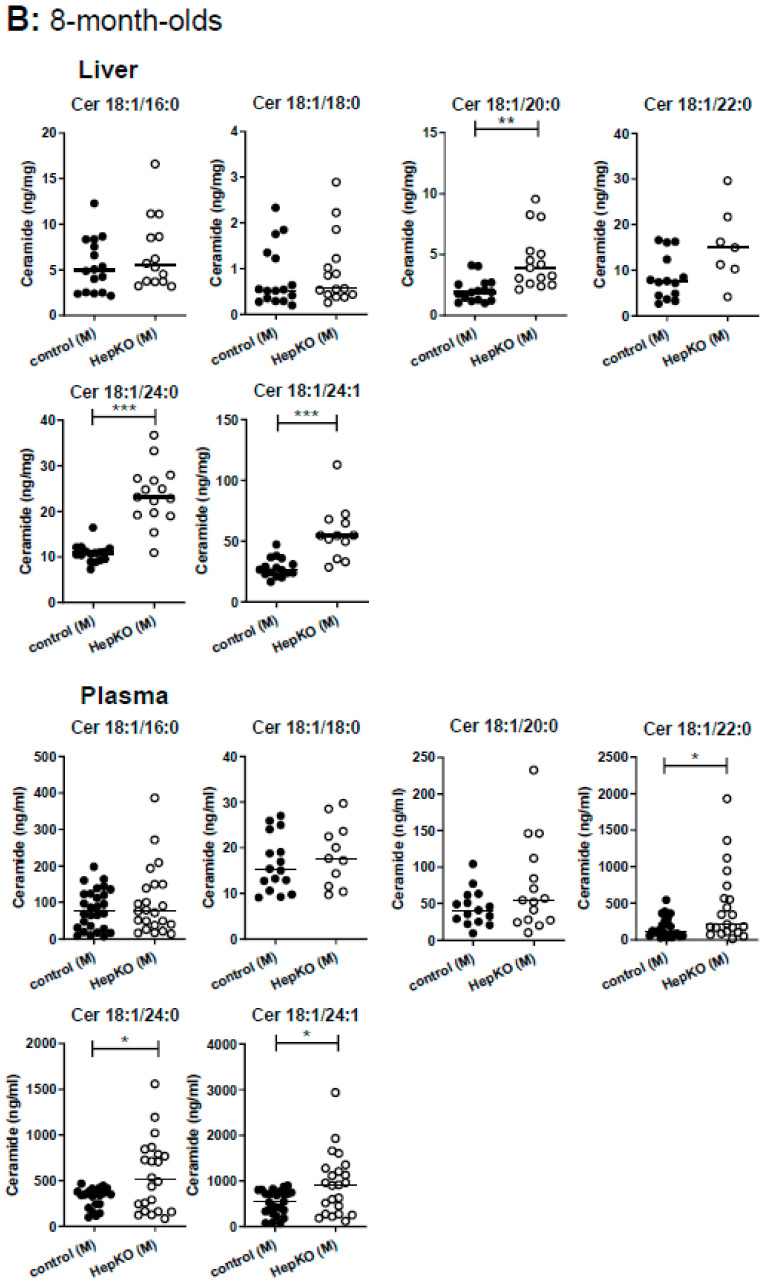
Levels of ceramides in liver and plasma of *Sgpl1*^HepKO^ mice. Ceramide (Cer) levels were measured by LC-MS/MS in liver and plasma of *Sgpl1*^control^ and *Sgpl1*^HepKO^ mice at the age of 8 weeks (**A**) or 8 months (**B**). Shown are values from individual mice and their median. * *p* < 0.05, ** *p* < 0.01, *** *p* < 0.001 in two-tailed Student’s *t*-test with Welch’s correction (**A**, liver: *n* = 13–21; **A**, plasma: *n* = 4–24; **B**, liver: *n* = 7–16; **B**, plasma: *n* = 11–28).

One such compensatory mechanism could be enhanced metabolism of S1P and sphingosine to other sphingolipids. Indeed, livers of 8-week-old *Sgpl1*^HepKO^ mice had about 2-fold elevated levels of all analyzed ceramide species, C18:1/16:0, C18:1/18:0 (except for the females which had higher levels of this ceramide species in general), C18:1/20:0, C18:1/24:0 and C18:1/24:1 (Figure 3A). Ceramides were significantly elevated also in the plasma of 8-week-old *Sgpl1*^HepKO^ mice (Figure 3A). Also in 8-month-old *Sgpl1*^HepKO^ mice, ceramides accumulated in both liver and plasma (Figure 3B). Measurements of glucosylceramides and sphingomyelin in liver and plasma of 8-month-old mice revealed that there were no significant alterations, except for C18:1/24:1 sphingomyelin which was increased in plasma of *Sgpl1*^HepKO^ mice (Figures S2 and S3).

**Figure 4:**
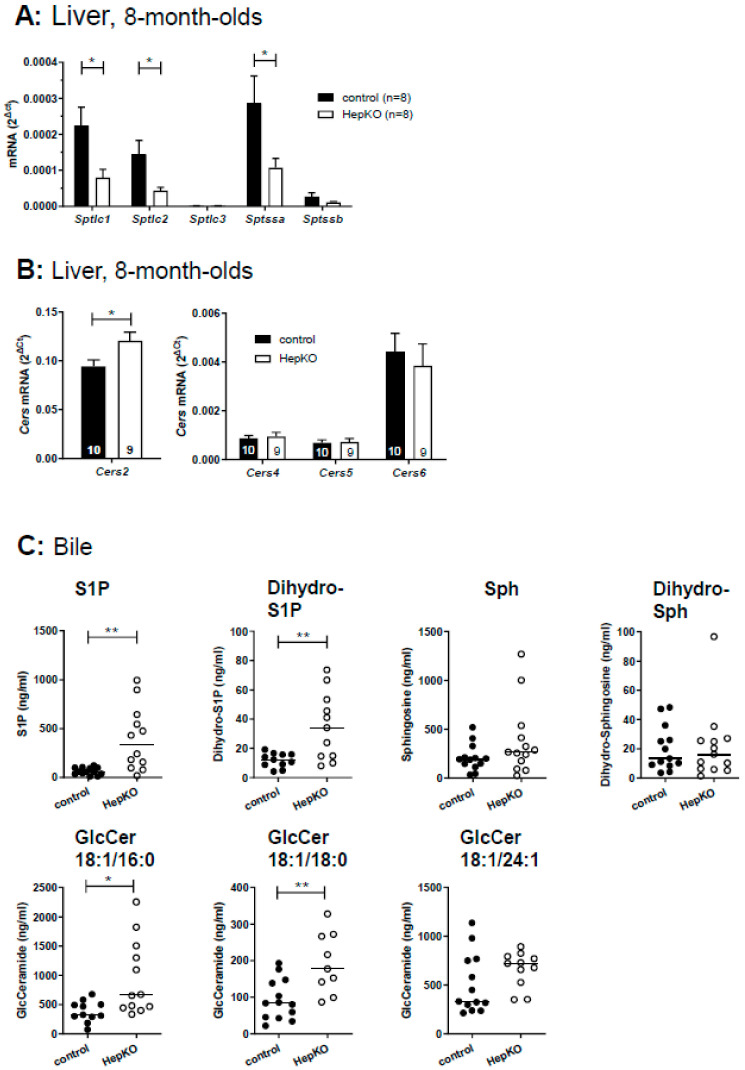
Expression of serine palmitoyltransferase subunits and ceramide synthases in liver of *Sgpl1*^HepKO^ mice, and secretion of sphingolipids into bile. (**A**,**B**) mRNA levels of different subunits of serine palmitoyltransferase (**A**) and *Cers* isoforms (**B**) were determined by quantitative real-time PCR in liver homogenates of *Sgpl1*^control^ and *Sgpl1*^HepKO^ mice at the age of 8 months. Data are means ± SEM of the indicated number of individual mice. * *p* < 0.05 in two-tailed Student’s *t*-test. (**C**) Levels of S1P, sphingosine (Sph) and glucosylceramides (GlcCer) were measured by LC-MS/MS in bile of *Sgpl1*^control^ and *Sgpl1*^HepKO^ mice at the age of 8 months. Shown are values from individual mice and their median (*n* = 9–13). * *p* < 0.05, ** *p* < 0.01 in two-tailed Student’s *t-*test with Welch’s correction.

In cells lacking SGPL1, de novo sphingolipid synthesis via serine palmitoyl transferase may be suppressed, leading to decreases in dihydro-sphingolipids lacking the 4,5 double bond [27,28]. Indeed, we observed a significant downregulation of mRNA of the serine palmitoyl transferase subunits, *Sptlc1*, *Sptlc2* and *Sptssa*, in liver of *Sgpl1*^HepKO^ mice (Figure 4A). Nevertheless, dihydro-S1P and dihydro-sphingosine were increased in livers of 8-week-old female *Sgpl1*^HepKO^ mice (Figure S4A,B), and dihydro-S1P was increased in livers of 8-month-old male mice (Figure S4C). Neither dihydro-S1P nor dihydro-sphingosine were significantly altered in plasma of *Sgpl1*^HepKO^ mice (Figure S4A–D). Furthermore, the dihydro-ceramides within the detection range were unaltered, except for C18:0/24:1 ceramide, which in fact was significantly decreased in livers of 8-week-old female and 8-month old male *Sgpl1*^HepKO^ mice (Figure S4E,F). This situation resembles that in liver of global *Sgpl1* knockout mice, in which mRNA of *Sptlc1* was reduced while dihydro-S1P and dihydro-sphingosine were elevated [18]; dihydro-ceramides were not shown in that study. Although it may be that expression but not activity of serine palmitoyl transferase was reduced in liver of global and hepatocyte-specific *Sgpl1* knockouts, increases in dihydro-S1P and dihydro-sphingosine despite reduced de novo sphingolipid synthesis have been observed also in other cell types lacking *Sgpl1*, for example neurons [27]; the reason for this remains unclear.

Since the defect in S1P degradation led to accumulation of ceramides, we wondered whether *Cers* expression was altered in *Sgpl1*^HepKO^ mice. Analysis of *Cers* mRNA revealed that expression of the predominant *Cers* isoform, *Cers*2, was slightly enhanced (by ~1.3-fold) in *Sgpl1*^HepKO^ mice, while *Cers*4–6 were not altered (Figure 4B). *Cers*1 and *Cers*3 were only weakly expressed (data not shown). In conclusion, in livers of *Sgpl1*^HepKO^ mice, constitutively expressed *Cers* isoforms may convert the accumulating S1P and sphingosine to ceramides which are then secreted into plasma and can probably be degraded elsewhere.

Hepatocytes express a high number of efflux transporters for secretion of diverse molecules into blood or bile, respectively [29,30]. Therefore, we applied LC-MS/MS to analyze whether the sphingolipid content of bile was altered in *Sgpl1*^HepKO^ mice. Indeed, deletion of *Sgpl1* in hepatocytes led to a strong increase in S1P and dihydro-S1P in bile of these mice (Figure 4C). Remarkably, sphingosine, dihydro-sphingosine and diverse ceramides were not significantly altered (Figure 4C and Figure S5). In addition to S1P, only glucosylceramides with chain lengths of C18:1/16:0 and C18:1/18:0 were significantly elevated in bile of *Sgpl1*^HepKO^ mice (Figure 4C), suggesting that there is a preference of this excretion pathway for S1P and glucosylceramides.

**Figure 5:**
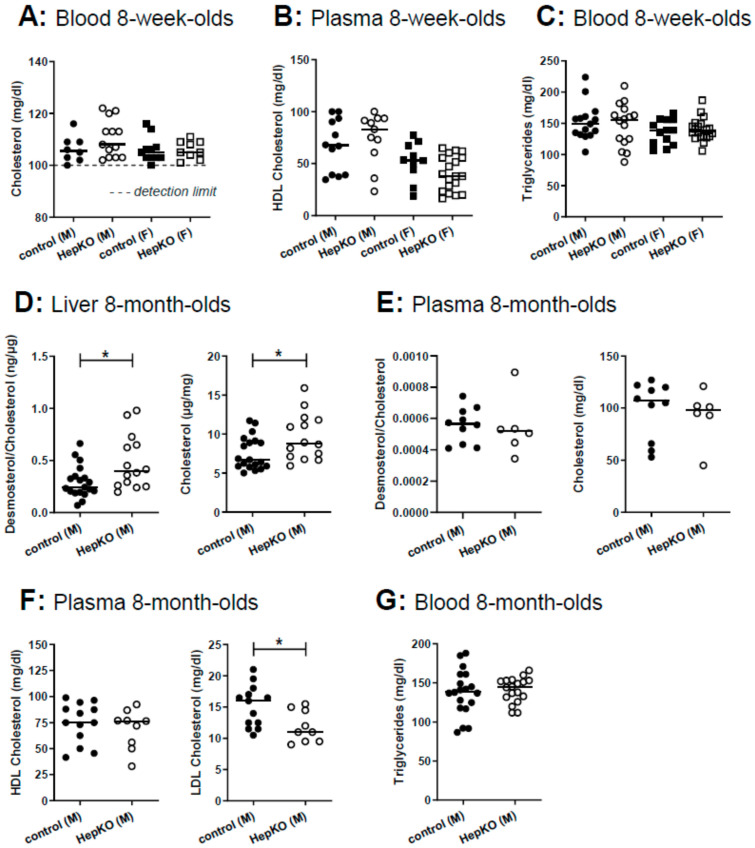
Cholesterol and triglyceride content in liver and plasma of *Sgpl1*^HepKO^ mice. Total cholesterol (whole blood, **A**), HDL cholesterol (plasma, **B**) and triglycerides (whole blood, **C**) were measured in 8 week old mice using a Reflotron analyser. (**D**–**G**) Cholesterol and triglycerides in 8 month old mice. (**D**,**E**) Cholesterol and its precursor desmosterol were determined by gas chromatography-flame ionization detection and gas chromatography-mass spectrometry, respectively, in liver (**D**) and plasma (**E**). Desmosterol is expressed as fold cholesterol. (**F**) HDL and LDL cholesterol in plasma were determined photometrically. (**G**) Triglycerides were determined in whole blood using a Reflotron analyser. Shown are values from individual mice and their median (**A**, *n* = 8–13; **B**, *n* = 10–19; **C**, *n* = 13–20; **D**, *n* = 14 and 19; **E**, *n* = 6 and 10; **F**, *n* = 9 and 13; **G**, *n* = 18 and 19). * *p* < 0.05 in two-tailed Student’s *t*-test.

In global *Sgpl1* knockout mice, not only sphingolipids but also cholesterol accumulated in liver and serum [18]. Therefore, we measured cholesterol and triglycerides in blood, and HDL cholesterol in plasma of 8-week-old mice, but there were no differences between controls and *Sgpl1*^HepKO^ mice (Figure 5A–C). However, a comprehensive analysis of sterols in the liver of 8-month-old *Sgpl1*^HepKO^ mice revealed that cholesterol and its immediate precursor, desmosterol, were significantly increased (Figure 5D). In plasma of 8-month-old *Sgpl1*^HepKO^ mice, cholesterol and desmosterol were not altered, similar to the younger animals (Figure 5E). Other sterols were normal in liver and plasma of 8-month-old *Sgpl1*^HepKO^ mice (Figure S6A,B). HDL cholesterol and triglycerides in plasma and blood, respectively, were not altered in 8-month-old *Sgpl1*^HepKO^ mice, similar to the younger animals (Figure 5F,G). Interestingly, LDL cholesterol, which is less abundant than HDL cholesterol in mice [31], was significantly decreased in 8-month-old *Sgpl1*^HepKO^ mice (Figure 5F).

**Figure 6:**
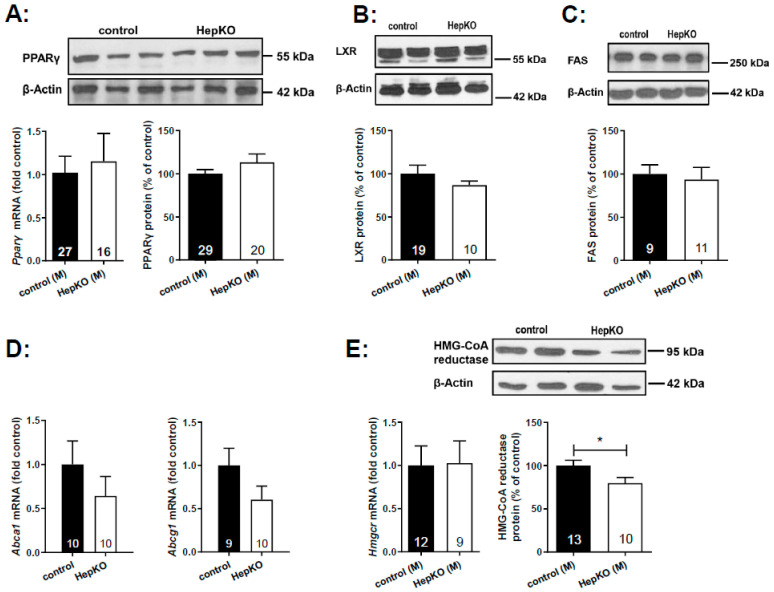

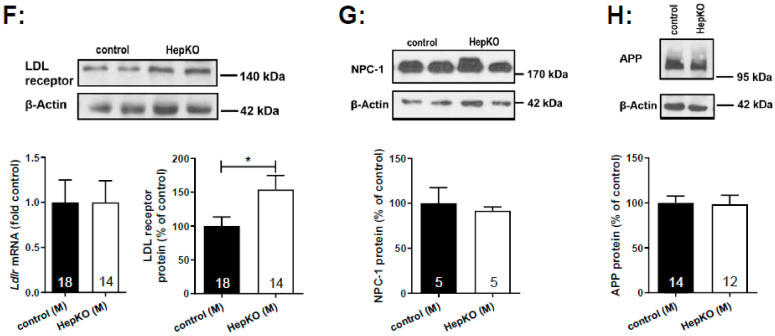
Expression of genes and proteins involved in lipid metabolism in liver of *Sgpl1*^HepKO^ mice. Expression of mRNA and protein levels was analyzed by quantitative real-time PCR and Western blotting, respectively, in liver homogenates of male *Sgpl1*^control^ (black bars) and *Sgpl1*^HepKO^ mice (white bars) at the age of 8 months. (**A**) PPARγ; (**B**) liver X receptor (LXR); (**C**) fatty acid synthase (FAS); (**D**) *Abca1* and *Abcg1*; (**E**) HMG-CoA reductase; (**F**) LDL receptor; (**G**) NPC1; (**H**) APP. Shown are representative blots from individual mice, and densitometric quantification of blots of the indicated number of mice. All values are means ± SEM. * *p* < 0.05 in two-tailed Student’s *t*-test.

Expression of key enzymes of cholesterol and lipid homeostasis was analyzed by quantitative polymerase chain reaction (PCR) and Western blot in liver homogenates of 8-month-old mice (Figure 6). Bektas et al. had shown a ~3.5-fold upregulation of *Pparγ* mRNA in the liver of global *Sgpl1* knockout mice [18]. However, hepatocyte-specific deletion of *Sgpl1* did not alter mRNA or protein expression of PPARγ in the liver (Figure 6A). Similarly, protein expression of liver X receptor was unaltered (Figure 6B). Furthermore, expression of LXR target genes such as fatty acid synthase (protein; Figure 6C), and the cholesterol transporters, *Abca1* and *Abcg1* (mRNA; Figure 6D) were not significantly altered. Western blot analysis revealed a slight decrease in HMG-CoA reductase protein (Figure 6E), despite the increased concentrations of the cholesterol precursor molecule, desmosterol, described above. 

Interestingly, the LDL receptor was upregulated in livers of *Sgpl1*^HepKO^ mice (Figure 6F), matching the decreased LDL cholesterol and increased liver cholesterol content of these mice. mRNA levels of *Hmgcr* and *Ldlr* were not different between *Sgpl1*^HepKO^ and *Sgpl1*^control^ mice (Figure 6E,F), indicating that their expression was regulated at the post-translational level. In MEF lacking *Sgpl1*, we had shown an accumulation of cholesterol in endo-/lysosomes with similarity to NPCD, which went along with increased expression of APP [24]. However, neither NPC-1 (Figure 6G) nor APP (Figure 6H) were regulated in liver of *Sgpl1*^HepKO^ mice.

**Figure 7:**
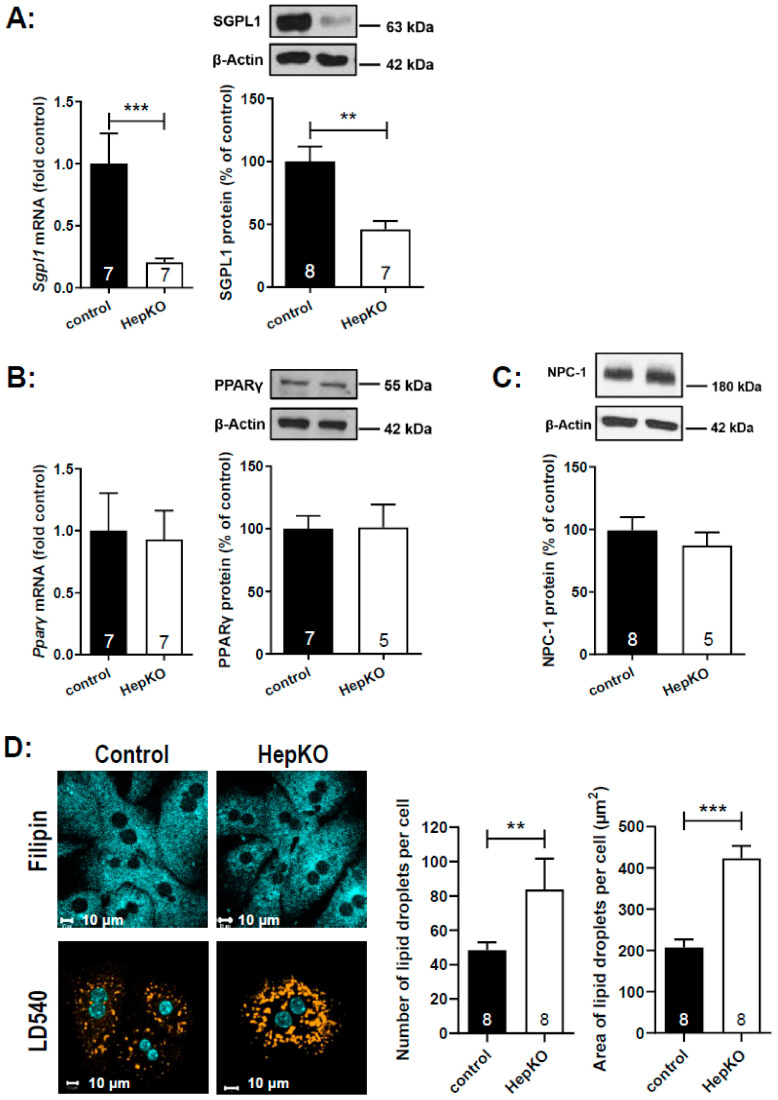
Characteristics of isolated hepatocytes of *Sgpl1*^HepKO^ mice. (**A**–**C**) Expression of mRNA and protein levels was determined by quantitative real-time PCR and Western blotting, respectively, in hepatocytes isolated from male *Sgpl1*^control^ (black bars) and *Sgpl1*^HepKO^ mice (white bars). Shown are representative blots of hepatocyte lysates of individual mice, and densitometric quantification of blots of the indicated number of mice. All values are means ± SEM. ** *p* < 0.05, *** *p* < 0.001 in nonparametric Mann-Whitney test (mRNA) or two-tailed Student’s *t*-test (protein). (**D**) Hepatocytes of *Sgpl1*^control^ and *Sgpl1*^HepKO^ mice were stained with filipin (cholesterol), LD540 (neutral lipids) and DAPI (DNA), as indicated. Bars, 10 µm. Quantification of the number of lipids droplets per cell was performed with ImageJ, using images from eight mice per group as detailed in the Section 4.

For further functional analysis, we isolated hepatocytes from *Sgpl1*^control^ and *Sgpl1*^HepKO^ mice. Expression of *Sgpl1* mRNA and SGPL1 protein was strongly reduced in these primary cell cultures, but not zero (Figure 7A). This is likely due to contamination of hepatocyte cultures with other cell types (not shown). Similar to the liver homogenates, mRNA and protein expression of PPARγ (Figure 7B) and protein expression of NPC-1 (Figure 7C) was not altered in hepatocytes from *Sgpl1*^HepKO^ mice. In search for a NPCD-like phenotype, we stained the hepatocytes with the cholesterol-binding dye, filipin. However, we did not see any cholesterol sequestration in hepatocytes from *Sgpl1*^HepKO^ mice (Figure 7D). On the other hand, staining of neutral lipids with LD540 revealed that *Sgpl1*-deficient hepatocytes had ~1.7-fold more lipid droplets than control hepatocytes (Figure 7D). The area covered by droplets was ~200 µm^2^ per cell in control hepatocytes, but ~400 µm^2^ per cell in *Sgpl1*-deficient hepatocytes (Figure 7D). Although we did not analyze the content of the droplets, it may be speculated that part of the accumulating ceramides was stored there as acyl-ceramides [32].

## 3. Discussion

We show here that in contrast to the global deletion of *Sgpl1* in mice, specific deletion of *Sgpl1* in mouse hepatocytes caused a mild phenotype with normal body weight, liver weight, liver histology and liver enzymes at least until the age of 8 months. Also, expression of key enzymes such as PPARγ and liver X receptor (including its target genes, fatty acid synthase, *Abca1* and *Abcg1*) was unaltered in *Sgpl1*^HepKO^ mice. Concentrations of S1P, sphingosine and ceramides in liver of *Sgpl1*^HepKO^ mice were only mildly elevated, on average, by ~1.5–2-fold, and sphingomyelin was not altered. In contrast, in liver of global *Sgpl1* knockout mice, S1P was elevated by >400-fold and sphingosine by ~40-fold [18]. Ceramides with C14, C16, C18 and C18:1 fatty acid chains were increased by ~10-fold, and also sphingomyelins were elevated in the global knockouts [18]. This huge difference easily explains the mild changes we observed in *Sgpl1*^HepKO^ mice, including the lack of *Pparγ* induction, which in global *Sgpl1* knockout mice was increased by ~3.5-fold on mRNA level [18]. Interestingly, C20, C24 and C24:1 ceramides were not altered in the global knockout, whereas in *Sgpl1*^HepKO^ liver, all measured C18:1 ceramides, with C16, C18, C20, C22, C24 and C24:1 acyl chains, were increased in 8-week-old mice, and there was even a preference for C18:1/C20, C18:1/C24 and C18:1/C24:1 ceramides in 8-month-old mice. Bektas et al. suggested that the exceedingly high S1P concentrations in the global *Sgpl1* knockout inhibited CerS2 via its S1P binding motif [33], thereby suppressing the formation of the very long chain ceramides [18]. In our system, S1P levels were probably not high enough for effective inhibition of CerS2, which was even slightly induced on mRNA level. However, the specific decrease in the very long chain dihydro-ceramide, C18:0/24:1 ceramide, might be due to a mild inhibition of CerS2 activity. The elevation of very long chain ceramides with a double bond might then be due to strongly enhanced flux from S1P via sphingosine to ceramides despite mild reduction of CerS2 activity in our system.

The mild phenotype of *Sgpl1*^HepKO^ mice is likely due to the fact that hepatocytes lacking *Sgpl1* can secrete the accumulating sphingolipids into blood and bile, so that they can be degraded by SGPL1 expressed in other cell types or eliminated via the intestinal route. In fact, several sphingolipids were elevated in plasma of *Sgpl1*^HepKO^ mice, but again, not as high and as extensive as in serum of the global knockout. Here, it remains unclear which of the increased sphingolipid molecules were primarily secreted from the liver, and which were generated by metabolic conversions within the vascular system or by distant organs. In particular, C18:1/C24 and C18:1/C24:1 ceramides, along with C18:1/C24:1 sphingomyelin, were steadily elevated in plasma, and we assume that sphingolipids containing these fatty acid chains are metabolically more stable than the others.

Most interestingly, plasma S1P was not elevated. S1P is remarkably stable in blood *in vitro*, but *in vivo*, its half time in blood is as short as ~15 min [34,35]. Kharel et al. have suggested that the liver is the primary site of blood S1P clearance [36]. Starting with the observation that *Sphk2*-deficient mice had elevated levels of plasma S1P [37], they used a hepatotropic adenovirus encoding Cre recombinase to delete *Sphk2*, phospholipid phosphatase-3, or *Sgpl1* from the liver of the respective floxed mice. Since deletion of these enzymes independently increased blood and plasma S1P, they suggested a model in which plasma S1P was dephosphorylated at the surface of hepatocytes by phospholipid phosphatase-3, followed by uptake of sphingosine, rephosphorylation by SphK2 and degradation by SGPL1 [36]. Most importantly, intravenous application of hepatotropic adenovirus encoding Cre recombinase into *Sgpl1^fl/fl^* mice significantly elevated blood and plasma S1P at 7–10 days after injection. In the present study, however, we observed that *Sgpl1* in hepatocytes was not required to keep plasma S1P in a normal range. This may be due to a number of reasons. First of all, the time points were different: while Kharel et al. measured plasma S1P at 7–10 days after injection of the virus, we analyzed plasma S1P in 8-week-old and 8-month-old mice. Thus, compensatory mechanisms had more time to develop in our system. Such a compensatory mechanism might be conversion of excess plasma S1P into sphingosine and further into ceramides. Sphingosine in fact was elevated in our mice at the age of 8 weeks, but not 8 months, indicating that compensatory mechanisms developed during ageing. Again, it is unclear whether this plasma sphingosine originated from S1P in plasma or liver. Another compensatory mechanism could be upregulation of hepatic transporters for excretion of S1P in bile. Furthermore, the methods that were applied are different. It is likely that intravenous application of hepatotropic adenovirus caused expression of Cre recombinase and thereby deletion of *Sgpl1* not only in hepatocytes but also in other cell types. Cells providing SGPL1 to compensate its loss in hepatocytes might be endothelial cells, since deletion or overexpression of SGPL1 in HUVEC increased or decreased [^3^H]S1P, respectively, in the supernatants [35]. The role of hepatocytes for clearance of plasma S1P is furthermore challenged by observations that hepatectomy resulted in the reduction of plasma S1P levels in mice [38]. Finally, at least ApoM-bound S1P could be eliminated via the LDL receptor. Thus, LDL receptor overexpressing mice had reduced S1P and ApoM in plasma [39]. Interestingly, the LDL receptor was upregulated in liver of *Sgpl1*^HepKO^ mice, and there was evidence for enhanced uptake of LDL cholesterol into liver (see below). This route, however, would require further processing of S1P in the liver via metabolism or its excretion into bile.

Indeed, S1P was elevated in bile of *Sgpl1*^HepKO^ mice. The ABC transporters, ABCA1, ABCC1, ABCC4, and ABCG2 [40,41,42,43]), spinster-2 and Mfsd2b have been shown to transport S1P (reviewed in [8,9]). ABCB4/MDR3 is the relevant transporter for excretion of the most abundant lipid in bile, phosphatidylcholine, while ABCB11 mediates bile salt secretion, ABCG5–8 sterol excretion, and ABCB1 and ABCC2 excretion of organic anions and diverse drugs [44]. Which transporter mediates S1P excretion into bile remains to be discovered. Importantly, our data do not preclude that hepatocytes secrete S1P also into plasma, since other cells types may contribute to its clearance from the circulation, as described above. Of note, it has been reported that conjugated bile acids activate the S1P receptor, S1P_2_ [45]. Our results suggest that when studying this effect, the content of S1P in bile acid preparations must be thoroughly analyzed. In contrast to S1P, ceramides were not elevated in bile of *Sgpl1*^HepKO^ mice, although C18:1/C14, C18:1/C16, C18:1/C18, C18:1/C20, C18:1/C22, C18:1/C24 and C18:1/C24:1 ceramides were present in mouse bile. Thus, excess ceramides generated as a consequence of hepatocyte *Sgpl1* deletion are obviously secreted exclusively into blood, probably as components of lipoproteins [46]. Glucosylceramides, on the other hand, were elevated in bile of *Sgpl1*^HepKO^ mice, indicating that indeed there was enhanced formation of glucosylceramides in hepatocytes lacking *Sgpl1*, although they were not increased in liver homogenates of these mice.

In global *Sgpl1* knockout mice, Bektas et al. have described a massive increase in serum phospholipids, triacylglycerol and cholesterol. Also in liver of these mice, diacylglycerol, triacylglycerol and cholesterol esters were elevated [18]. Diacylglycerol was suggested to be a by-product of enhanced sphingomyelin synthesis [18], and since we did not observe elevation of sphingomyelins, we did not address this otherwise important point. However, we have described previously that mouse embryonic fibroblasts from *Sgpl1* knockout mice had a severe disturbance of cholesterol homeo-stasis. In these cells, cholesterol accumulated in endo/lysosomes, and lack of cholesterol at the endoplasmic reticulum activated SREBP2 and subsequently upregulated the LDL receptor [24]. This vicious cycle resembled that in NPCD, in which cholesterol is trapped in endo/lysosomes due to defective NPC proteins which mediate cholesterol export from these organelles (reviewed in [47]). This prompted us to analyze cholesterol homeostasis in *Sgpl1*^HepKO^ mice. We show here that cholesterol and its precursor, desmosterol, were elevated in liver by ~1.3-fold and ~1.6-fold, respectively, as net result from a slight downregulation of HMG-CoA reductase and upregulation of the LDL receptor. In plasma, total and HDL-associated cholesterol were normal, but LDL cholesterol was decreased, in agreement with enhanced LDL uptake via the upregulated LDL receptor. The results from plasma thus oppose those in global *Sgpl1* knockout mice and human SPLIS patients, which may have hypercholesterolemia [12]. Furthermore, we did not detect any sign of an NPCD-like phenotype as cholesterol was evenly distributed in hepatocytes, expression of NPC-1 was unaltered and secondary effects such as APP induction were not observed. Finally, the LDL receptor and HMG-CoA reductase were altered only on protein, not on mRNA level. This is important since it suggests that there was no local lack of cholesterol at the endoplasmic reticulum, triggering *Ldlr* and *Hmgcr* transcription via SREBP transcription factors, as it is the case in NPCD [47]. Protein expression of the LDL receptor is regulated by several pathways, including the E3 ubiquitin ligase IDOL (“inducible degrader of the LDL receptor”), the deubiquitylase USP2, or the proprotein convertase subtilisin/kexin type 9 [48,49,50]. It may be speculated that these pathways are modulated by one or more of the accumulating lipids in *Sgpl1*^HepKO^ mouse liver. A reason for the increase in desmosterol might be reduced activity of 3β-hydroxysterol-Δ^24^-reductase (DHCR24), which converts desmosterol to cholesterol and may be suppressed by high cholesterol [51,52]. Desmosterol activated the liver X receptor in macrophage foam cells from *Ldlr* knockout mice fed a high fat/high cholesterol diet [52], but this was not observed in mouse liver lacking DHCR24 [53]. In agreement, we did not see enhanced transcription of liver X receptor target genes. Thus, the regulation and role of elevated desmosterol in *Sgpl1*^HepKO^ mouse liver remains to be resolved.

Taken together, our data show that deletion of *Sgpl1* causes a highly cell type-specific accumulation of S1P and upstream sphingolipids, due to cell type-specific expression of sphingolipid metabolizing enzymes and transporters. The hepatocyte, particularly rich in diverse efflux pumps, is able to eliminate the accumulating sphingolipids to a large part, keeping liver function stable. *Sgpl1* in hepatocytes is not required for maintenance of plasma S1P. Finally, the role of S1P in bile, with its potential influence on the gut, remains an interesting question for future studies. These results have important implications for understanding the role of the liver in whole-body sphingolipid metabolism.

## 4. Materials and Methods

### 4.1. Materials

DPBS, HBSS, l-glutamine, penicillin/streptomycin, non-essential amino acids (NEAA), and William’s medium E were purchased from Gibco/Thermo Fisher Scientific (Darmstadt, Germany). Filipin III from streptomyces filipinensis, *N,N*-dimethylformamide, insulin, collagenase and TRI Reagent were obtained from Sigma Aldrich Chemie GmbH (Taufkirchen, Germany). Mayer’s Haematoxylin Solution was from AppliChem GmbH (Darmstadt, Germany). Eosin and DAPI were purchased from Carl Roth GmbH (Karlsruhe, Germany). Heparin sodium salt was from Ratiopharm (Ulm, Germany). LD540 was kindly provided by Prof. Dr. Christoph Thiele (University of Bonn, Life & Medical Sciences Institute, Bonn, Germany). All other materials were from previously described sources [24].

### 4.2. Generation and Analysis of Hepatocyte-Specific Sgpl1 Knockout Mice

All animal handling was performed in accordance with the German Animal Welfare Law and had been declared to the Animal Welfare Officer of the University. The animal housing facility was licensed by the local authorities (Ref. No. 32.62.1 completed by V57—19c 20/21 I—FU). The methods used to euthanize the animals were consistent with the recommendations of the AVMA Guidelines for the Euthanasia of Animals. Mice were kept under standard pathogen-free conditions with a 12:12 h day-night cycle and food supply ad libitum.

*Sgpl1^fl/fl^* mice, with exons 10–12 of *Sgpl1* flanked by loxP sites, have been described before [54]. They were crossed with mice expressing the Cre recombinase under control of the albumin promoter (B6.Cg-*Speer6-ps1^Tg(Alb-cre)21Mgn^*/J; Jax stock #003574 from The Jackson Laboratory; [55]). The offspring was backcrossed to obtain *Sgpl1*^fl/fl^/*AlbCre*^−/−^ (*Sgpl1*^control^ or control) or *Sgpl1*^fl/fl^/*AlbCre*^+/−^ (*Sgpl1*^HepKO^) mice. For genotyping, genomic DNA was extracted with REDExtract-N-Amp Tissue PCR Kit from Sigma Aldrich Chemie GmbH. The following primers were used: *Sgpl1*^fl/fl^, 5′-GTGGTTCTGGATGGAGTTTA-3′, 5′-GAAATTGAGCATATCCGTTC-3′ and 5′-TTGAGGCTTGTAAGGTTAAGTC-3′; *AlbCre*, 5′-GAAGCAGAAGCTTAGGAAGATGG-3′, 5′-TGCAAACATCACATGCACAC-3′ and 5′-TTGGCCCCTTACCATAACTG-3′. The mice were anaesthetized with isoflurane and euthanized by cervical dislocation. Then, they were weighed and blood was obtained by heart puncture. Blood samples were collected in heparin-coated vessels. Plasma samples were prepared by centrifugation of blood that was collected in EDTA-coated tubes (10 min, 2.000× *g*, 4 °C). Organs were perfused with DPBS (2.7 mM KCl, 1.5 mM KH_2_PO_4_, 138 mM NaCl, 8 mM Na_2_HPO_4_) supplemented with 2% heparin sodium salt before removal. Bile was taken by gall bladder puncture. ALT, AST, triglycerides and cholesterol were measured in whole blood, HDL cholesterol was measured in plasma with the Reflotron Plus blood analysis system (Roche Diagnostics, Mannheim, Germany). Blood cell counts were analyzed with the Hemovet scil Vet abc analyzer (Scil Animal Care Company GmbH, Viernheim, Germany). HDL and LDL cholesterol were measured with the Cobas 8000 c701 auto analyzer (Roche Diagnostics, Mannheim, Germany) by LABOKLIN GmbH & Co. KG (Bad Kissingen, Germany).

### 4.3. Histology

Paraffin-embedded liver sections were deparaffinized and rehydrated before incubating the tissue with Mayer’s Haematoxylin Solution (1 g/L haematoxylin) followed by 0.2% eosin. H&E-stained liver sections were analyzed with the Zeiss Axioskop 2 (Carl Zeiss Micro Imaging GmbH, Göttingen, Germany).

### 4.4. Isolation of Mouse Hepatocytes

The mice were anesthetized with isoflurane and euthanized by cervical dislocation. The liver was perfused with HBSS without CaCl_2_ and MgCl_2_ (5.3 mM KCl, 0.4 mM KH_2_PO_4_, 4.2 mM NaHCO_3_, 138 mM NaCl, 0.34 mM Na_2_HPO_4_, 5.6 mM glucose) supplemented with 15 mM HEPES, 2.5 mM EGTA, 5.5 mM glucose, 1xNEAA and 1% penicillin/streptomycin, followed by HBSS with CaCl_2_ and MgCl_2_ (1.3 mM CaCl_2_, 0.5 mM MgCl_2_, 0.4 mM MgSO_4_, 5.3 mM KCl, 0.4 mM KH_2_PO_4_, 4.2 mM NaHCO_3_, 138 mM NaCl, 0.34 mM Na_2_HPO_4_, 5.6 mM glucose) supplemented with 15 mM HEPES, 1xNEAA and 0.13 mg/mL collagenase from *Clostridium histolyticum* (Sigma #C9891). Afterwards, the liver was swivelled in William’s medium E supplemented with 10% FCS, 2 mM glutamine and 1% penicillin/streptomycin to flush the hepatocytes out of the liver. The cell suspension was squeezed through a 100 µm cell strainer (Thermo Fisher Scientific), washed twice with medium (centrifugation at 50× *g*, 5 min) and seeded onto cell culture plates.

### 4.5. Immunocytochemistry and Fluorescence Microscopy

Cholesterol in hepatocytes was stained with filipin III. The filipin III solution was prepared as described before [24]. The cells were washed twice with DPBS and fixed with 10% paraformaldehyde for 1 h on ice. Then, they were washed again three times with DPBS and incubated in the dark at room temperature for 1 h with 45 µM filipin III. Finally, the cells were washed three times with DPBS. Lipid droplets were stained with LD540. Cells were washed with HBSS and incubated with DAPI (1 µg/mL) for 10 min. 0.1 µg/mL LD540 was added for further 10 min. Then, the cells were washed again with HBSS. Confocal laser scanning microscopy was performed with a Zeiss LSM510 Meta system equipped with an inverted Observer Z1 microscope and a Plan-Apochromat 63×/1.4 oil immersion objective (Carl Zeiss MicroImaging GmbH) as described [24]. The following excitation (ex) laserlines and emission (em) filter sets were used: DAPI: ex 405 nm, em band-pass 420–480 nm; filipin III: ex 405 nm, em long-pass 420 nm; LD540: ex 543, em band-pass 560–615. Quantitative evaluation of lipid droplets in isolated hepatocytes (Figure 7D) was performed with ImageJ (version 1.52a). Spatial calibration of the images was done with the “set scale” function. The images were converted to 8 bit, thresholds were adjusted, and lipid droplets were quantified using the “analyze particles” function. Five images per mouse and eight mice per group (~15 hepatocytes per mouse) were evaluated.

### 4.6. Quantitative Real-Time PCR

Frozen mouse liver was homogenized with a Mikro-Dismembrator S (B. Braun Biotech International, Berlin, Germany) in TRI Reagent. RNA was isolated using a standardized phenol-based method. RNA was quantified using a NanoDrop spectrophotometer (Thermo Fisher Scientific) and transcribed into cDNA using a RevertAid First Strand cDNA Synthesis Kit (Applied Biosystems/Thermo Fisher Scientific) according to the manufacturer’s instructions. Quantitative real-time PCR was performed with Applied Biosystems 7500 Fast Real-Time PCR System. The following TaqMan probes were used: *Abca1* (Mm00442646_m1), *Abcg1* (Mm00437390_m1), *Hmgcr* (Mm01282499_m1), *Ldlr* (Mm01177349_m1), *Pparγ* (Mm01184322_m1), *Sgpl1* (Mm00627244_m1), *Sptlc1* (Mm00447343_m1), *Sptlc2* (Mm00448871_m1), *Sptlc3* (Mm01278138_m1), *Sptssa* (Mm01267361_g1), *Sptssb* (Mm01952615_u1), and *18s* (Hs99999901_s1); all labelled with FAM on the 5′ end. The mRNA expression levels of *Cers1*, *Cers2*, *Cers3*, *Cers4*, *Cers5*, *Cers6* (for primers, see [56]) and glyceraldehyde-3-phosphate dehydrogenase *(Gapdh)* (forward, 5′ aggtcggtgtgatttg-3′; reverse, 5′ tgtagaccataggtc-3′) were measured using Syber Select MasterMix with the QuantStudioTM5 Real Time PCR instrument. All PCR materials were obtained from Thermo Fisher Scientific.

### 4.7. Western Blotting

Frozen mouse liver was homogenized with a Mikro-Dismembrator S (B. Braun Biotech International) in lysis buffer (50 mM Tris-HCl pH 7.4, 150 mM NaCl, 50 mM NaF, 20 mM Na_4_P_2_O_7_, 2 mM EDTA, 2 mM EGTA, 2 mM DTT, 200 µM Na_3_VO_4_, 40 mM β-glycerolphosphate, 10% glycerol, 1% Triton X-100), separated by SDS gel electrophoresis and blotted onto polyvinylidene difluoride membranes. SGPL1 was probed with antibody HPA021125 from Atlas Antibodies (Bromma, Sweden). Amyloid precursor protein (ab32136), fatty acid synthase (ab128856), HMG-CoA reductase (ab174830), liver X receptor (ab176323), LDL receptor (ab30532), and NPC-1 (ab134113) antibodies were from Abcam (Cambridge, UK). For PPARγ, the antibody sc-7196 from Santa Cruz Biotechnology (Dallas, TX, USA) was used. Anti-β-actin (#A5441) was from Sigma Aldrich Chemie GmbH. Horseradish peroxidase-conjugated secondary antibodies were from GE Healthcare (Freiburg, Germany), and the enhanced chemiluminescence system was from Merck Millipore (Darmstadt, Germany).

### 4.8. Measurement of Sphingolipids except Sphingomyelins

The quantification of sphingolipids, except sphingomyelins, in plasma, bile fluid and liver tissue was performed by liquid chromatography tandem mass spectrometry (LC-MS/MS). The analytes were extracted by liquid-liquid extraction and analyzed using two different LC-MS/MS methods, one for sphingoid bases and the other one for ceramides. Further information can be found in the supplementary material.

### 4.9. Measurement of Sphingomyelins

In preparation for LC-MS/MS quantification, sphingomyelins were extracted from liver tissue and plasma as described previously [57]. Briefly, liver tissue was homogenized in aqueous buffered solution, and 20 µL aliquots of the homogenates (corresponding to tissue equivalents of 1 mg) or 10 µL plasma were subjected to lipid extraction with 1.5 mL methanol/chloroform (2:1, *v*:*v*). The extraction solvent contained C18:1/16:0-d_31_ sphingomyelin (Avanti Polar Lipids, Alabaster, AL, USA) as internal standard. Chromatographic separation was achieved on a 1260 Infinity HPLC (Agilent Technologies, Waldbronn, Germany) equipped with a Poroshell 120 EC-C8 column (3.0 × 150 mm, 2.7 μm; Agilent Technologies). An eluent system consisting of 0.1% formic acid in water (solvent A) and 0.1% formic acid in acetonitrile/methanol (1:1, *v*:*v*, solvent B), was used for gradient elution at an initial composition of 40:60 (A:B, *v*:*v*) and a flow rate of 0.5 mL/min. MS/MS analyses were carried out using a 6490 triple-quadrupole mass spectrometer (Agilent Technologies) operating in the positive electrospray ionization mode [58]. Six sphingomyelin (SM) sub-species were analyzed by multiple reaction monitoring. To this end, the following mass transitions were recorded (qualifier product ions in parentheses): *m/z* 703.6 → 184.1 (86.1) for C18:1/16:0 SM, *m/z* 731.6 → 184.1 (86.1) for C18:1/18:0 SM, *m/z* 734.8 → 184.1 (86.1) for C18:1/16:0-d_31_ SM, *m/z* 759.6 → 184.1 (86.1) for C18:1/20:0 SM, *m/z* 787.7 → 184.1 (86.1) for C18:1/22:0 SM, *m/z* 813.7 → 184.1 (86.1) for C18:1/24:1 SM, and *m/z* 815.7 → 184.1 (86.1) for C18:1/24:0 SM. Peak areas of sphingomyelin sub-species, as determined with MassHunter software (Agilent Technologies), were related to that of the internal standard (C18:1/16:0-d_31_ SM), followed by external calibration in the range of 1 to 50 pmol on column. Hepatic sphingomyelin contents were normalized to protein (as determined by Bradford assay) and expressed as pmol/mg protein. Plasma sphingomyelin levels were calculated as pmol/mL.

### 4.10. Measurement of Cholesterol, Non-Cholesterol Sterols and Oxysterols

Cholesterol in plasma and liver was determined by gas chromatography-flame ionization detection using 5α-cholestane as internal standard. Plasma and hepatic non-cholesterol sterols and oxysterols were determined by gas chromatography-mass spectrometry using epicoprostanol and deuterium labelled oxysterols as internal standards [59].

### 4.11. Statistical Analysis and Data Presentation

Graphical presentations and statistical analyses were performed with GraphPad Prism (version 9.1.0; GraphPad Software, San Diego, CA, USA). Statistical tests were used as indicated in the Figure Legends. For comparison of sphingolipid concentrations in *Sgpl1*^control^ and *Sgpl1*^HepKO^ mice, Student’s *t*-test with Welch’s correction was applied, since variances were usually higher in *Sgpl1*^HepKO^ mice. Microscopic images were analyzed and presented with the ZEN software (Carl Zeiss MicroImaging GmbH). For quantitative evaluation of Western blots, bands were analyzed with ImageJ (version 1.52a), normalized to β-actin and expressed as percent of *Sgpl1*^control^ mice. For evaluation of quantitative PCR, the cycle threshold (Ct) values were analyzed using Softmax V.5.4.6 from Molecular Devices (San Jose, CA, USA), except for *Cers* isoforms, which were analyzed with QuantStudioTM Design & Analysis Software V1.4.3 (both from Applied Biosystems/Thermo Fisher Scientific). Data were evaluated using the ∆∆Ct method, normalized to *18s* or *Gapdh* as housekeeping genes and expressed as fold of *Sgpl1*^control^ mice.

## Supplementary Materials

The following materials are available online https://www.mdpi.com/article/10.3390/ijms221910617/s1.

## Abbreviations

DHCR24: 3β-hydroxysterol-Δ^24^-reductase; ABC transporter, ATP-binding cassette transporter; ALT, alanine aminotransferase; ApoM, apolipoprotein M; APP, amyloid precursor protein; AST, aspartate aminotransferase; *Cers*, ceramide synthase mRNA; CerS, ceramide synthase protein; *Gapdh*, glyceraldehyde-3-phosphate dehydrogenase mRNA; GPCR, G-protein-coupled receptor; H&E, haematoxylin and eosin; HDL, high density lipoprotein; *Hmgcr*, 3-hydroxy-3-methylglutaryl-coenzyfigureme A reductase mRNA; HMG-CoA, 3-hydroxy-3-methylglutaryl-coenzyme A; LC-MS/MS, liquid chromatography tandem mass spectrometry; LDL, low density lipoprotein; *Ldlr*, LDL receptor mRNA; Mfsd2b, major facilitator superfamily domain-containing protein-2B; NEAA, non-essential amino acids; NPCD, Niemann-Pick disease type C; PCR, polymerase chain reaction; *Pparγ*, peroxisome proliferator-activated receptor-γ mRNA; PPARγ, peroxisome proliferator-activated receptor-γ protein; S1P, sphingosine-1-phosphate; *Sgpl1*, S1P lyase gene or mRNA; SGPL1, S1P lyase protein; *Sgpl1*^control^, *Sgpl1^fl/fl^/AlbCre^−/−^* (control) mice; *Sgpl1*^HepKO^, *Sgpl1^fl/fl^/AlbCre^+/−^* (hepatocyte specific *Sgpl1* knockout) mice; *Sphk*, sphingosine kinase gene or mRNA; SphK, sphingosine kinase protein; SPLIS, S1P lyase insufficiency syndrome; SREBP2, sterol regulatory element-binding protein-2; VLDL, very low density lipoprotein.
