# Supplementary material for: Mouse Liver Compensates Loss of Sgpl1 by Secretion of Sphingolipids into Blood and Bile
Source: Int J Mol Sci. 2021 Sep 30;22(19):10617. doi: 10.3390/ijms221910617 (PMC8508615; doi:10.3390/ijms221910617)
Supplement: Supplementary file 1 [file ijms-22-10617-s001.zip › Supplementary Figures.pdf]

Legends to Supplementary Figures

Suppl. Figure S1: Basic characteristics of *Sgpl1*$^{\mathbf{HepKO}}$ mice.

(A, B) Body weight and liver weight of control and *Sgpl1*^{HepKO} mice at the age of 8 weeks (A) and 8 months (B). (C, D) Liver enzymes and (E, F) Blood cell counts. Shown are values from individual mice and their median. M, male mice; F, female mice.

Suppl. Figure S2: Levels of glucosylceramides in liver and plasma of *Sgpl1*$^{\mathbf{HepKO}}$ mice.

Glucosylceramides were measured by LC-MS/MS in liver and plasma of control and *Sgpl1*^{HepKO} mice at the age of 8 months. Shown are values from individual mice and their median. GlcCer, glucosylceramide.

Suppl. Figure S3: Levels of sphingomyelins in liver and plasma of *Sgpl1*$^{\mathbf{HepKO}}$ mice.

Sphingomyelins (SM) were measured by LC-MS/MS in liver and plasma of control and *Sgpl1*^{HepKO} mice at the age of 8 months. Shown are values from individual mice and their median. *$p<0.05$ in two-tailed Student's t-test with Welch's correction.

Suppl. Figure S4: Levels of dihydro-sphingolipids in liver and plasma of *Sgpl1*$^{\mathbf{HepKO}}$ mice.

(A-D) Dihydro-S1P and dihydro-sphingosine were measured by LC-MS/MS in liver and plasma of control and *Sgpl1*^{HepKO} mice at the age of 8 weeks (A, B) and 8 months (C, D). (E, F) Dihydro-ceramides levels were measured in liver at the age of 8 weeks (E) and 8 months (F). Shown are values from individual mice and their median. *$p<0.05$, **$p<0.01$, ***$p<0.001$ in two-tailed Student's t-test with Welch's correction.

Suppl. Figure S5: Levels of ceramides in bile of *Sgpl1*$^{\mathbf{HepKO}}$ mice.

Ceramide levels were measured by LC-MS/MS in bile of control and *Sgpl1*^{HepKO} mice at various ages. Shown are values from individual mice and their median.

Suppl. Figure S6: Sterols in liver and plasma of *Sgpl1*$^{\mathbf{HepKO}}$ mice.

(A, B) Sterols referred to total cholesterol were measured by gas chromatography-flame ionisation detection in the liver (A) and plasma (B) of control and *Sgpl1*^{HepKO} mice at the age of 8 months. Shown are values from individual mice and their median.

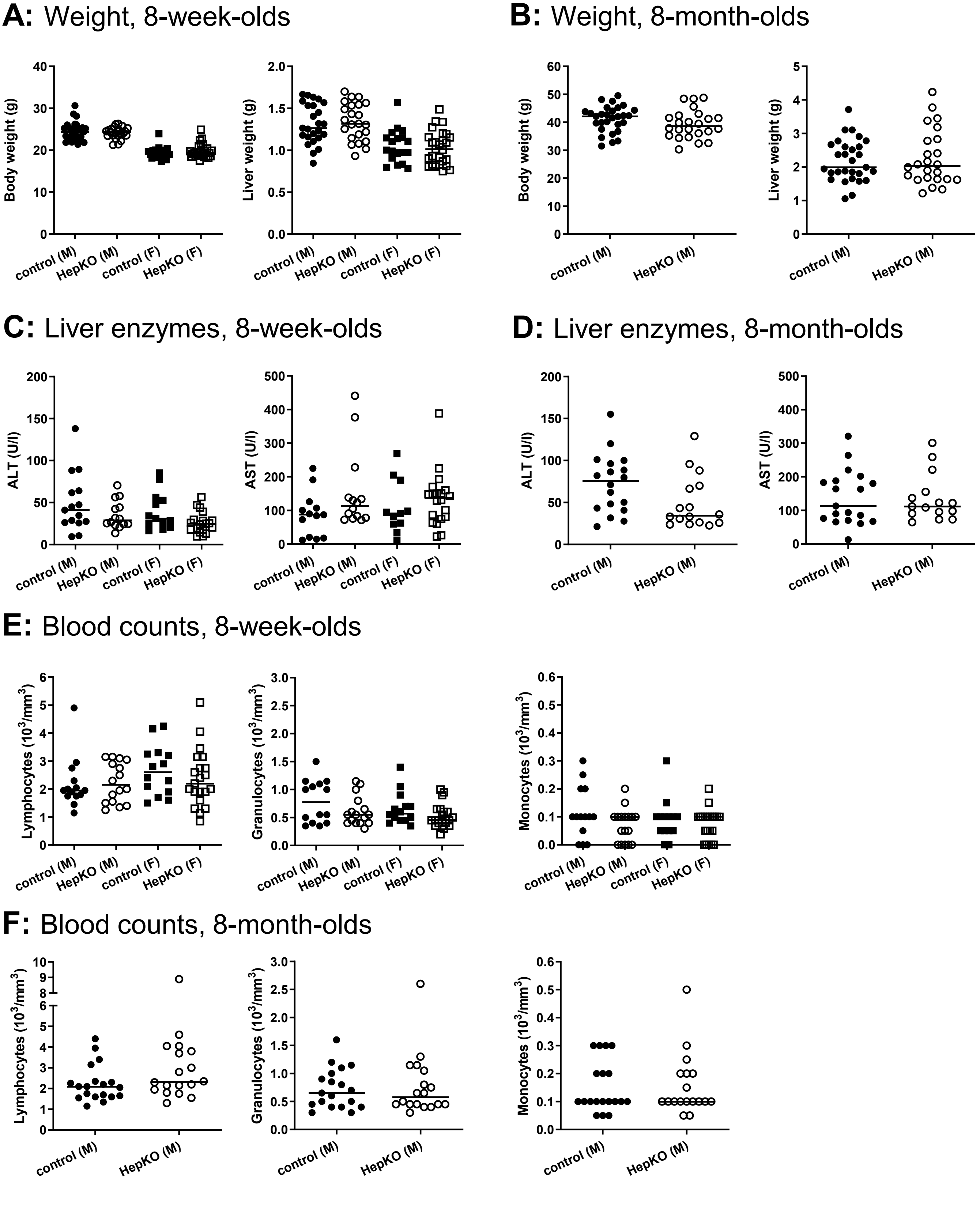
A: Weight, 8-week-olds
B: Weight, 8-month-olds
Body weight (g)
Liver weight (g)
control (M)
HepKO (M)
control (F)
HepKO (F)
C: Liver enzymes, 8-week-olds
D: Liver enzymes, 8-month-olds
ALT (U/l)
AST (U/l)
E: Blood counts, 8-week-olds
F: Blood counts, 8-month-olds
Lymphocytes (10^3/mm^3)
Granulocytes (10^3/mm^3)
Monocytes (10^3/mm^3)

Figure S1

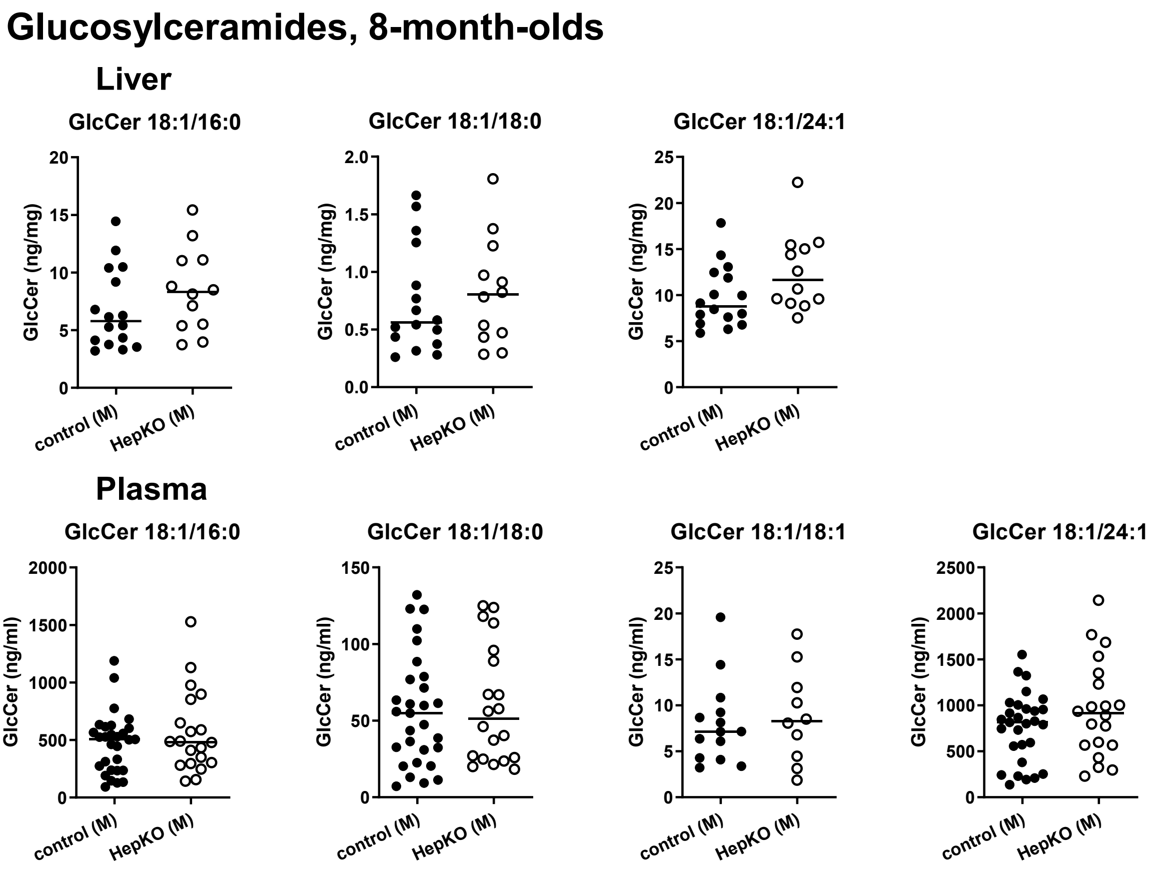
Glucosylceramides, 8-month-olds
Liver
GlcCer 18:1/16:0
GlcCer 18:1/18:0
GlcCer 18:1/24:1
GlcCer (ng/mg)
control (M)
HepKO (M)
Plasma
GlcCer 18:1/16:0
GlcCer 18:1/18:0
GlcCer 18:1/18:1
GlcCer 18:1/24:1
GlcCer (ng/ml)
control (M)
HepKO (M)

Figure S2

Sphingomyelin, 8-month-olds

Liver

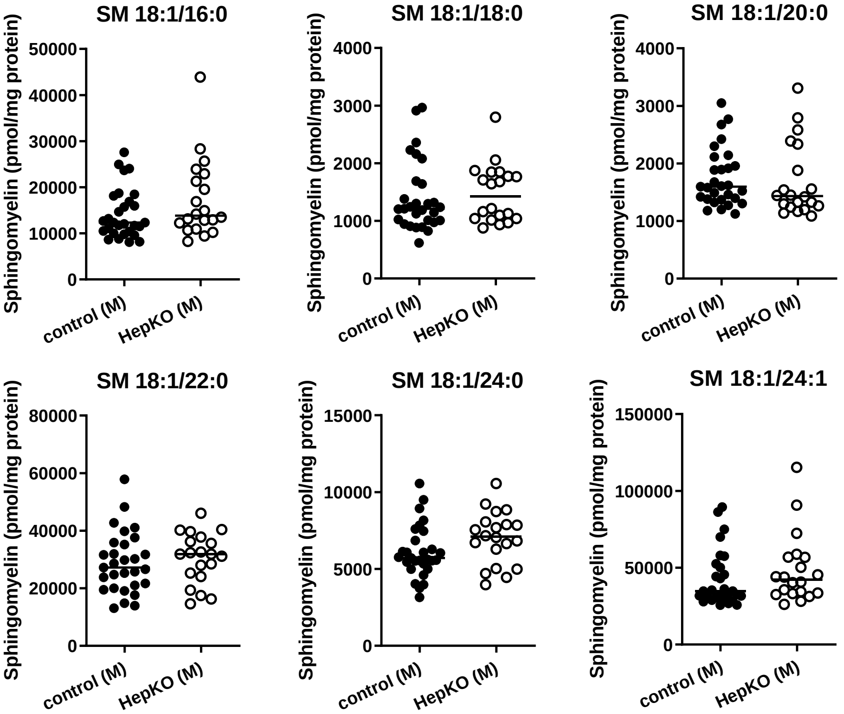

Plasma

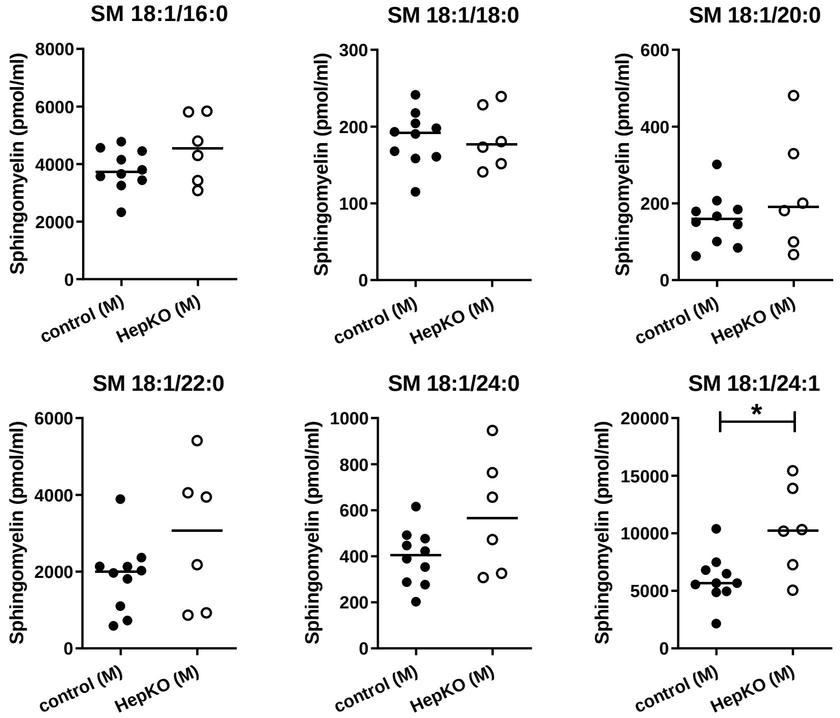

Figure S3

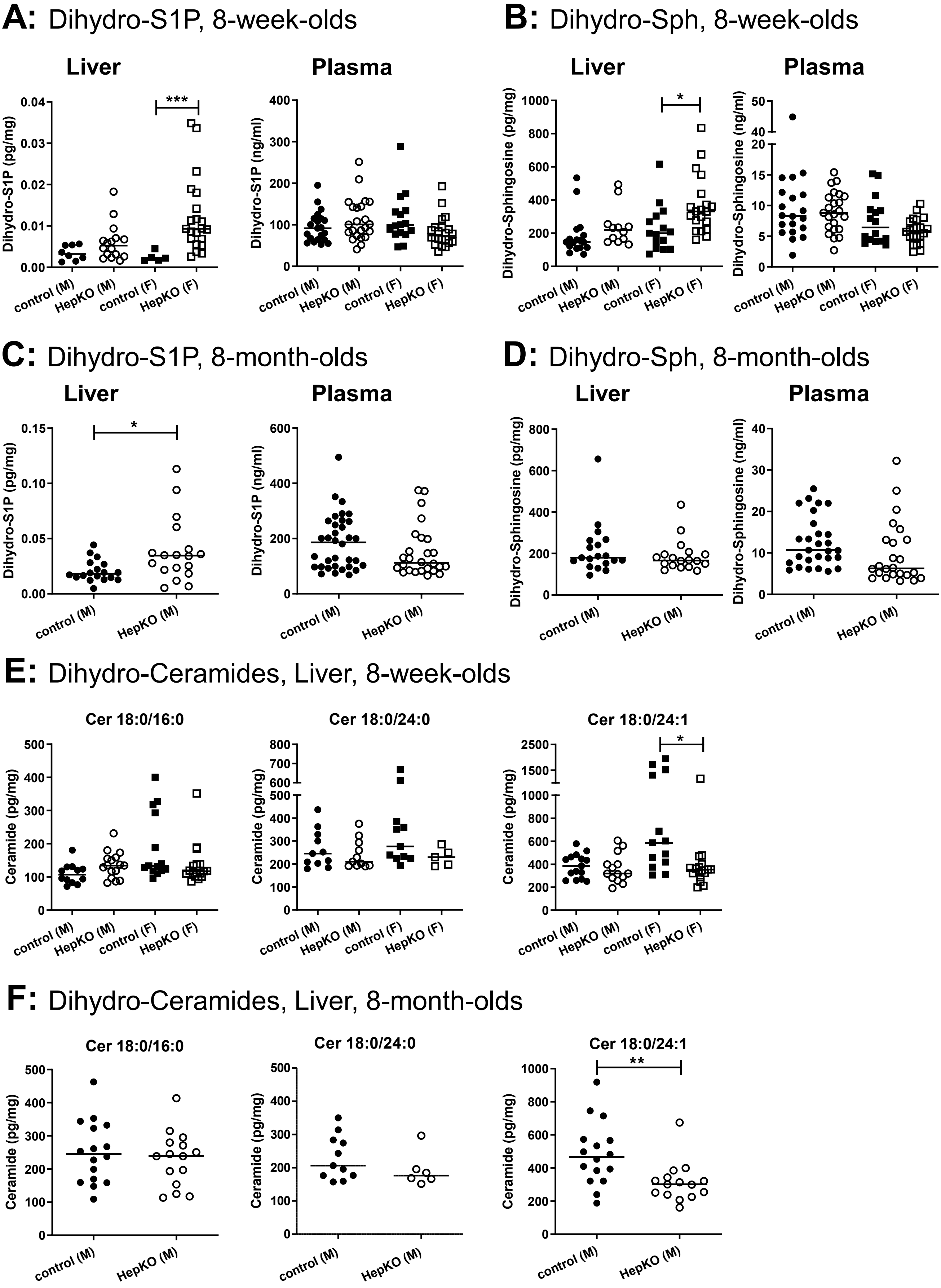
A: Dihydro-S1P, 8-week-olds
Liver
Plasma
Dihydro-S1P (pg/mg)
Dihydro-S1P (ng/ml)
B: Dihydro-Sph, 8-week-olds
Liver
Plasma
Dihydro-Sphingosine (pg/mg)
Dihydro-Sphingosine (ng/ml)
C: Dihydro-S1P, 8-month-olds
Liver
Plasma
Dihydro-S1P (pg/mg)
Dihydro-S1P (ng/ml)
D: Dihydro-Sph, 8-month-olds
Liver
Plasma
Dihydro-Sphingosine (pg/mg)
Dihydro-Sphingosine (ng/ml)
E: Dihydro-Ceramides, Liver, 8-week-olds
Cer 18:0/16:0
Cer 18:0/24:0
Cer 18:0/24:1
Ceramide (pg/mg)
F: Dihydro-Ceramides, Liver, 8-month-olds
Cer 18:0/16:0
Cer 18:0/24:0
Cer 18:0/24:1
Ceramide (pg/mg)
control (M)
HepKO (M)
control (F)
HepKO (F)

Figure S4

Ceramides in Bile, 8-month-olds

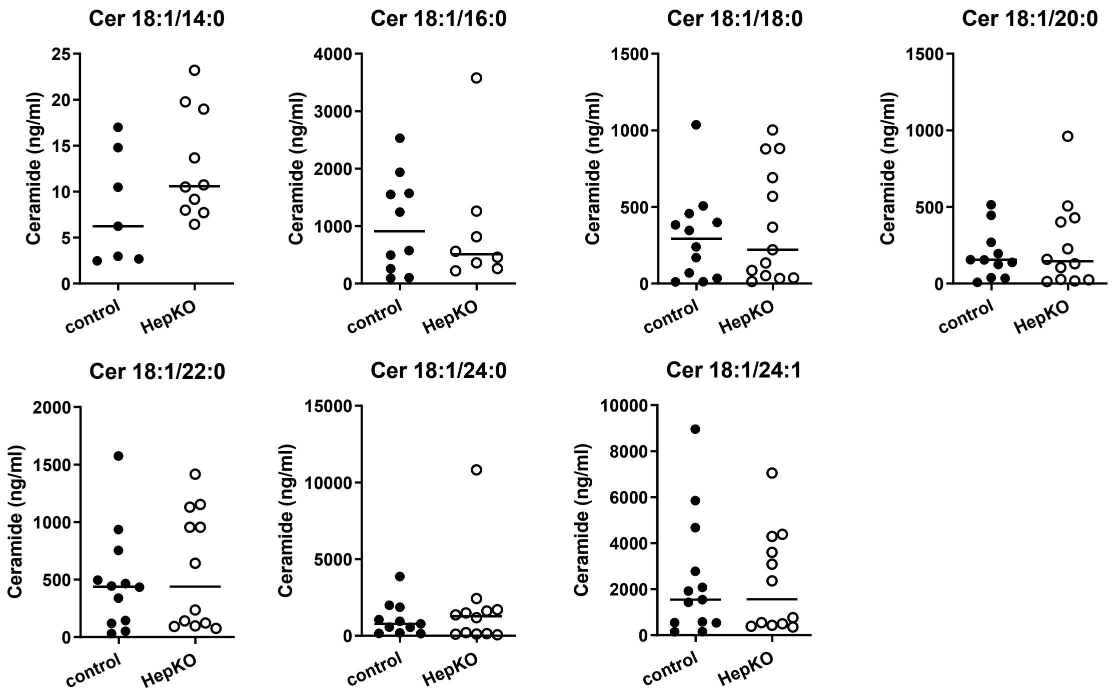

Figure S5

A: Sterols in liver, 8-month-olds

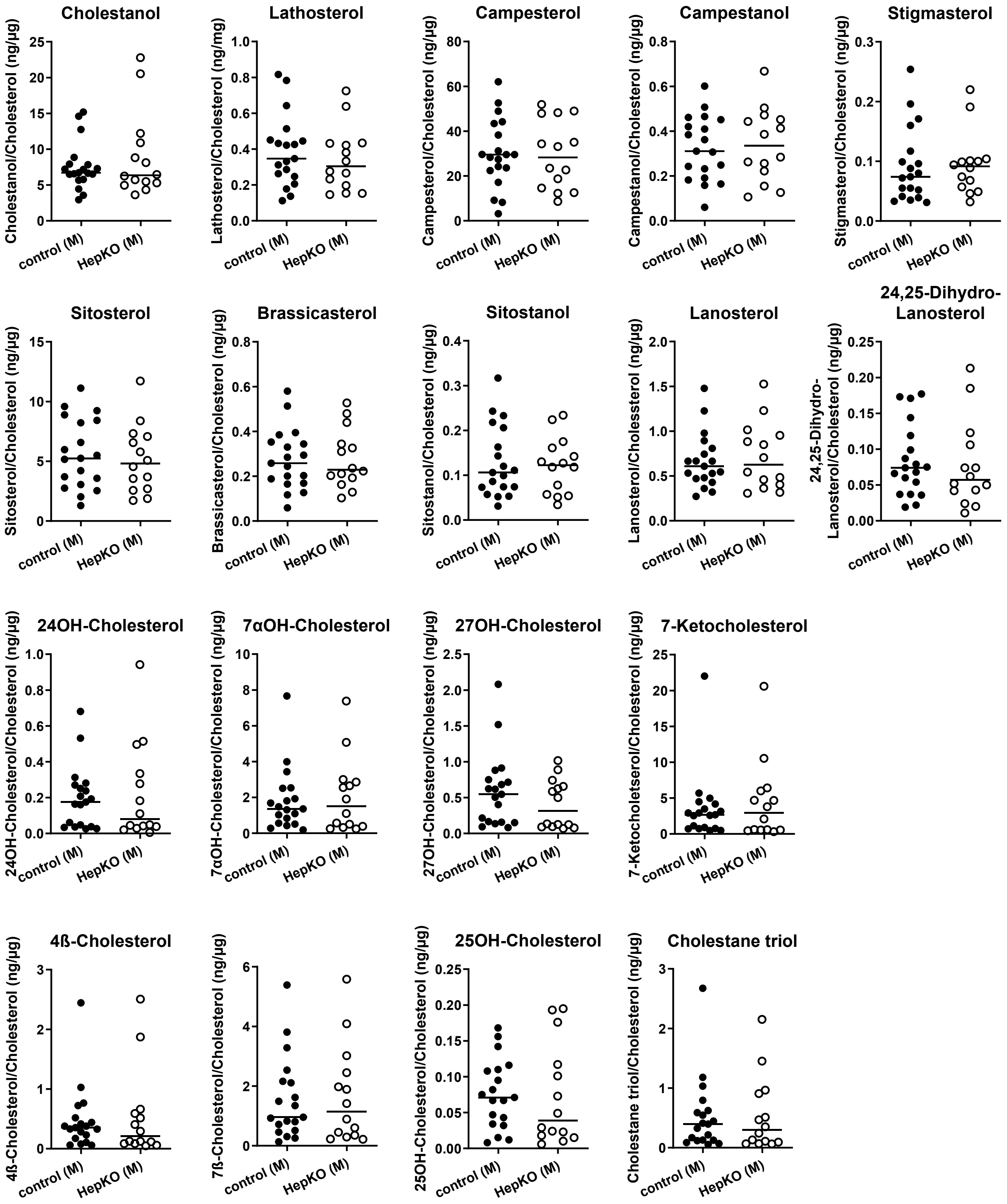

Figure S6

B: Sterols in plasma, 8-month-olds

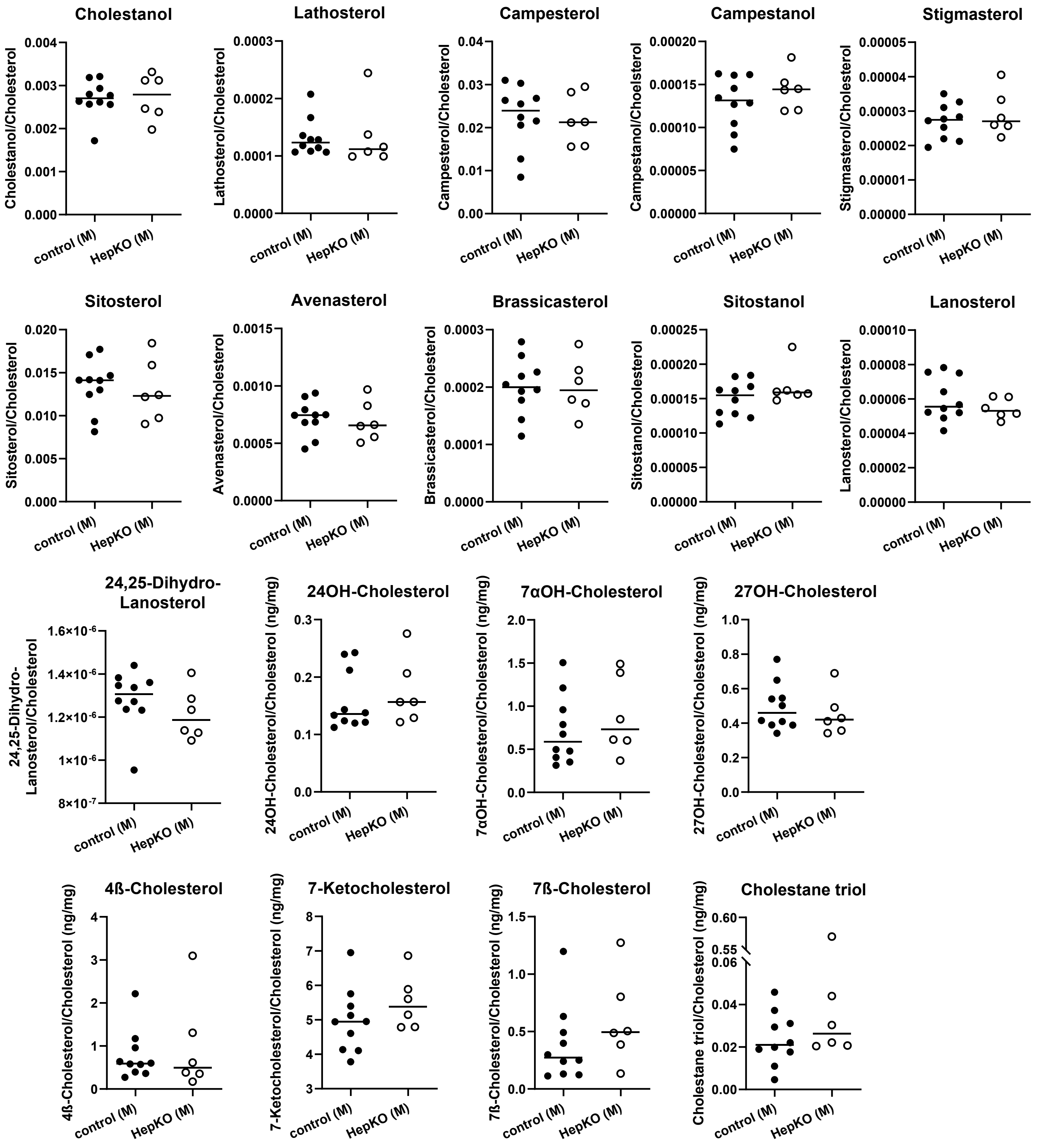

Figure S6 (cont.)
