# Supplementary material for: Mouse Liver Compensates Loss of Sgpl1 by Secretion of Sphingolipids into Blood and Bile
Source: Int J Mol Sci. 2021 Sep 30;22(19):10617. doi: 10.3390/ijms221910617 (PMC8508615; doi:10.3390/ijms221910617)
Supplement: Supplementary file 1 [file ijms-22-10617-s001.zip › Supplementary Methods.pdf]

Detailed method of sphingolipid measurements, except sphingomyelins, by liquid chromatography-tandem mass spectrometry

Reference substances

Sphingoid bases:

SPH d18:1 – Avanti Polar Lipids, Alabaster, AL, USA

SPH d18:0 – Avanti Polar Lipids, Alabaster, AL, USA

SPH d20:1 – Avanti Polar Lipids, Alabaster, AL, USA

SPH d20:0 – Avanti Polar Lipids, Alabaster, AL, USA

S1P d16:1– Avanti Polar Lipids, Alabaster, AL, USA

S1P d18:1– Avanti Polar Lipids, Alabaster, AL, USA

S1P d18:0 – Avanti Polar Lipids, Alabaster, AL, USA

Ceramides:

Cer d18:0/16:0 – Avanti Polar Lipids, Alabaster, AL, USA

Cer d18:0/18:0 – Avanti Polar Lipids, Alabaster, AL, USA

Cer d18:0/24:0 – Avanti Polar Lipids, Alabaster, AL, USA

Cer d18:0/24:1 – Avanti Polar Lipids, Alabaster, AL, USA

Cer d18:1/14:0 – Avanti Polar Lipids, Alabaster, AL, USA

Cer d18:1/16:0 – Avanti Polar Lipids, Alabaster, AL, USA

Cer d18:1/18:0 – Avanti Polar Lipids, Alabaster, AL, USA

Cer d18:0/18:1 – Avanti Polar Lipids, Alabaster, AL, USA

Cer d18:1/20:0 – Avanti Polar Lipids, Alabaster, AL, USA

Cer d18:1/22:0 – Avanti Polar Lipids, Alabaster, AL, USA

Cer d18:1/24:0 – Avanti Polar Lipids, Alabaster, AL, USA

Cer d18:1/24:1 – Avanti Polar Lipids, Alabaster, AL, USA

GlcCer d18:1/16:0 – Avanti Polar Lipids, Alabaster, AL, USA

GlcCer d18:1/18:0 – Avanti Polar Lipids, Alabaster, AL, USA

GlcCer d18:1/18:1 – Avanti Polar Lipids, Alabaster, AL, USA

GlcCer d18:1/24:1 – Avanti Polar Lipids, Alabaster, AL, USA

LacCer d18:1/16:0 – Avanti Polar Lipids, Alabaster, AL, USA

LacCer d18:1/18:0 – Avanti Polar Lipids, Alabaster, AL, USA

LacCer d18:1/18:1 – Avanti Polar Lipids, Alabaster, AL, USA

LacCer d18:1/24:0– Avanti Polar Lipids, Alabaster, AL, USA

LacCer d18:1/24:1 – Avanti Polar Lipids, Alabaster, AL, USA

Internal Standards:

SPH d18:1-d7 – Avanti Polar Lipids, Alabaster, AL, USA

SPH d18:0-d7 – Avanti Polar Lipids, Alabaster, AL, USA
S1P d18:1-d7 – Avanti Polar Lipids, Alabaster, AL, USA
S1P d18:0-d7 – Avanti Polar Lipids, Alabaster, AL, USA
Cer d18:0/18:0-d3 – Avanti Polar Lipids, Alabaster, AL, USA
Cer d18:0/24:0-d7 – Avanti Polar Lipids, Alabaster, AL, USA
Cer d18:1/16:0-d7 – Avanti Polar Lipids, Alabaster, AL, USA
Cer d18:1/18:0-d7 – Avanti Polar Lipids, Alabaster, AL, USA
Cer d18:1/24:0-d4 – Chiroblock GmbH, Wolfen, Germany
Cer d18:1/24:1-d7 – Avanti Polar Lipids, Alabaster, AL, USA
GlcCer d18:1/18:0-d5 – Avanti Polar Lipids, Alabaster, AL, USA
GlcCer d18:1/24:1-d5 – Avanti Polar Lipids, Alabaster, AL, USA
LacCer d18:1/16:0-d3 – Avanti Polar Lipids, Alabaster, AL, USA
LacCer d18:1/17:0 – Avanti Polar Lipids, Alabaster, AL, USA
LacCer d18:1/24:0-d7 – Avanti Polar Lipids, Alabaster, AL, USA

Sample preparation and extraction

Working solutions for the generation of calibrator and QC-samples of sphingoid bases were prepared as a mixture of all sphingoid bases by serial dilution using methanol. For ceramides, working solutions were prepared as a mixture of all ceramides by serial dilution using chloroform/methanol (2:1, v/v) with 0.1 % butylated hydroxytoluene. The working solution of the internal standard was prepared as a mixture of all internal standards using chloroform/methanol (2:1, v/v) with 0.1 % butylated hydroxytoluene. Plasma or bile fluid samples were thawed at room temperature. Liver tissue was homogenized using an appropriate volume of water/ethanol 3:1 (v/v), resulting in a tissue suspension with a concentration of 0.05 mg/µL. Cell pellets were resuspended in 200 µL extraction buffer (citric acid 30 mM, disodium hydrogen phosphate 40 mM).

Samples were processed as follows:

1. Take 10 µL plasma or 10 µL bile fluid or 20 µL tissue suspension (1 mg tissue) or 200 µL cell pellet sample.
2. Add 200 µL extraction buffer (citric acid 30 mM, disodium hydrogen phosphate 40 mM), except for cell pellet samples.
3. Add 20 µL internal standard solution.
4. Extract mixture once with 600 µL methanol/chloroform/hydrochloric acid (15:83:2, v/v/v).
5. Remove lower organic phase, split it in two aliquots and evaporate both aliquots at 45 °C under a gentle stream of nitrogen.
6. Reconstitute in

   A 50 µL of methanol/formic acid (95:5, v/v) for sphingoid base measurement.

B 50 µL of tetrahydrofuran/0.2% formic acid and 10 mM ammonium formate (9:1, v/v) for ceramide measurement.

For the preparation of calibration standards and quality control samples, 20 µL of a working solution were processed as stated instead of 10 µL sample. Quality control samples of three different concentration levels (low, middle, high) were run as initial and final samples of each run.

Instrumentation

A Sphingoid base measurements

Mass spectrometer QTrap 5500 (Sciex, Darmstadt, Germany)
Turbo-V-source in positive ESI mode

HPLC Agilent 1290 Infinity UHPLC system (Agilent, Waldbronn, Germany)
1290 BinPump, 1290 TCC SL, 1290 Sampler, 1290 Thermostat

LC-column Zorbax Eclipse Plus C8, 30 mm x 2.1 mm ID, 1.8 µm (Agilent technologies, Waldbronn, Germany) with corresponding precolumn

Solvent A 0.5 % formic acid
Solvent B acetonitrile/isopropanol/acetone/formic acid (50:30:19:1, v/v/v/v)

Injection volume 5 µL

Table S1: Gradient program for the separation of sphingoid bases.

Time	Flow Rate [µL]	Percent solvent A	Percent solvent B
0.0	400	55	45
0.5	400	55	45
1.5	400	0	100
3.0	400	0	100
3.1	400	55	45
4.5	400	55	45

After every sample injection, two injections of extraction buffer were run to avoid carry-over effects.

The analysis was done in Multiple Reaction Monitoring (MRM) mode. Information on the used precursor to product ion transitions (m/z) and internal standards can be found in Table 2. Data Acquisition was done using Analyst Software V 1.6.3 and quantification was performed with

MultiQuant Software 3.0.3 (both Sciex, Darmstadt, Germany), employing the internal standard method (isotope dilution mass spectrometry). Calibration curves were calculated by linear or quadratic regression with 1/x or 1/x2 weighting. Variations in accuracy were less than 15% over the whole range of calibration, except for the lower limit of quantification, where a variation in accuracy of 20% was accepted.

Table S2: Sphingolipids: precursor to product ion transitions (m/z) and internal standards.

Analyte	Q1	Q3 (Quantifier/Qualifier)	IS
SPH d18:1	300.3	252.3 / 282.3	SPH d18:1-d7
SPH d18:0	302.3	284.3 / 254.2	SPH d18:0-d7
SPH d20:1	328.3	310.1 / 280.1	SPH d18:1-d7
SPH d20:0	330.3	312.4 / 60.0	SPH d18:0-d7
S1P d16:1	352.2	236.2 / 334.2	S1P d18:1-d7
S1P d18:1	380.2	264.2 / 362.3	S1P d18:1-d7
S1P d18:0	382.3	284.1 / 266.3	S1P d18:0-d7

B Ceramide measurements

Mass spectrometer	QTrap 5500 (Sciex, Darmstadt, Germany)<br>Turbo-V-source in positive ESI mode
HPLC	Agilent 1290 Infinity UHPLC system (Agilent, Waldbronn, Germany)<br>1290 BinPump, 1290 TCC SL, 1290 Sampler, 1290 Thermostat
LC-column	Zorbax Eclipse Plus C18, 50 mm x 2.1 mm ID, 1.8 µm (Agilent technologies, Waldbronn, Germany) with corresponding precolumn
Solvent A	10 mM ammonium formate in water + 0.2 % formic acid
Solvent B	acetonitrile/isopropanol/acetone (50/30/20, v/v/v) + 0.2 % formic acid
Injection volume	5 µL

Table S3: Gradient program for the separation of ceramides.

Time	Flow Rate [µL]	Percent solvent A	Percent solvent B
0.0	350	50	50
0.2	350	50	50
0.6	350	10	90
4.0	350	0	100

5.5	350	0	100
6.0	350	50	50
7.5	350	50	50

The analysis was done in Multiple Reaction Monitoring (MRM) mode. Information on the used precursor to product ion transitions (m/z) and internal standards can be found in Table 4. Data Acquisition was done using Analyst Software V 1.6.3 and quantification was performed with MultiQuant Software 3.0.3 (both Sciex, Darmstadt, Germany), employing the internal standard method (isotope dilution mass spectrometry). Calibration curves were calculated by linear or quadratic regression with 1/x or 1/x2 weighting. Variations in accuracy were less than 15% over the whole range of calibration, except for the lower limit of quantification, where a variation in accuracy of 20% was accepted.

Table S4: Ceramides: precursor to product ion transitions (m/z) and internal standards.

Analyte	Q1	Q3 (Quantifier/Qualifier)	IS
Cer d18:0/16:0	540.5	284.3 / 522.5	Cer d18:0/18:0-d3
Cer d18:0/18:0	568.6	284.3 / 550.6	Cer d18:0/18:0-d3
Cer d18:0/24:0	652.7	284.2 / 634.2	Cer d18:0/24:0-d7
Cer d18:0/24:1	650.6	284.2 / 632.6	Cer d18:0/24:0-d7
Cer d18:1/14:0	510.5	264.2 / 492.5	Cer d18:1/16:0-d7
Cer d18:1/16:0	538.5	264.2 / 520.4	Cer d18:1/16:0-d7
Cer d18:1/18:0	566.5	264.4 / 548.5	Cer d18:1/18:0-d3
Cer d18:1/18:1	564,5	264.3 / 546.4	Cer d18:1/18:0-d3
Cer d18:1/20:0	594.6	264.4 / 576.5	Cer d18:1/18:0-d3
Cer d18:1/22:0	622.6	264.4 / 604.4	Cer d18:1/24:0-d4
Cer d18:1/24:0*	651.6	264.2 / 633.6	Cer d18:1/24:0-d4
Cer d18:1/24:1*	649.6	264.2 / 631.6	Cer d18:1/24:1-d7
GlcCer d18:1/16:0*	701.6	264.2 / 683.5	GlcCer d18:1/18:0-d5
GlcCer d18:1/18:0	728.6	264.2 / 710.6	GlcCer d18:1/18:0-d5
GlcCer d18:1/18:1	726.6	264.2 / 708.5	GlcCer d18:1/18:0-d5
GlcCer d18:1/24:1*	811.7	264.3 / 793.7	GlcCer d18:1/24:1-d7
LacCer d18:1/16:0*	863.6	264.3 / 521.5	LacCer d18:1/16:0-d3
LacCer d18:1/18:0	890.6	264.2 / 548.5	LacCer d18:1/17:0
LacCer d18:1/18:1	888.7	264.3 / 546.7	LacCer d18:1/17:0
LacCer d18:1/24:0	974.7	264.3 / 632.6	LacCer d18:1/24:0-d7
LacCer d18:1/24:1*	973.7	264.2 / 631.5	LacCer d18:1/24:0-d7

*because of high signal intensity the ^{13}C-isotope was measured.
